# Identification of Single Nucleotide Polymorphisms Through Genome-Wide Association Studies of pH Traits in Goose Meat

**DOI:** 10.3390/biology13110865

**Published:** 2024-10-24

**Authors:** Haiwei Wang, Zhuping Chen, Lin Ma, Yifan Wu, Xianzhi Zhao, Keshan Zhang, Jiajia Xue, Yi Luo, Chao Wang, Zuohua Liu, Youhui Xie, Ying Chen, Guangliang Gao, Qigui Wang

**Affiliations:** 1 Chongqing Engineering Research Center of Goose Genetic Improvement, Institute of Poultry Science, Chongqing Academy of Animal Science, Chongqing 402460, China; wahwe@163.com (H.W.); 2019102008@stu.sicau.edu.cn (Z.C.); 82101215414@caas.cn (L.M.); zhaoxianzhi2002@163.com (X.Z.); zhangksh1988@163.com (K.Z.); xuejiajia25@163.com (J.X.); homleestar@163.com (Y.L.); wangccq@foxmail.com (C.W.); liuzuohua66@163.com (Z.L.); xyh917111@163.com (Y.X.); chzhli75@163.com (Y.C.); 2 College of Animal Science and Technology, Southwest University, Chongqing 402460, China; wyf18723079024@outlook.com

**Keywords:** goose, pH, genetic parameters, SNP, genome-wide association study (GWAS)

## Abstract

The genetic regulation of goose meat quality traits remains relatively unexplored, and the underlying mechanisms are yet to be elucidated. This study aims to employ single nucleotide polymorphism (SNP) genotyping in conjunction with genome-wide association studies (GWAS) to investigate critical candidate regions and genes associated with the pH trait of meat in Sichuan white geese. A cohort of 203 healthy male Sichuan white geese was randomly selected and slaughtered at 70 days of age. Measurements were taken of meat pH, growth parameters, body dimensions, and post-slaughter traits. High-throughput sequencing on the Illumina HiSeq X Ten platform facilitated gene resequencing and SNP evaluation, and GWAS was employed to detect key genes within quantitative trait loci (QTL) intervals. The sequencing of 203 individuals yielded a total of 2601.19 Gb of genomic data, with an average sequencing depth of 10.89×. Through GWAS analysis, a total of 30 SNPs associated with pH were identified. These SNPs were identified on multiple chromosomes, including on chromosome 17 (chr: 23.57–23.68 Mb) and chromosome 13 (chr13: 31.52–31.61 Mb). By annotating these associated SNPs, nine candidate genes (including *C19L2, AMFR, POL, RERGL, ZN484, GMDS, WAC*) associated with the pH of goose meat were identified. The matrix-assisted laser desorption/ionization time-of-flight mass spectrometry (MALDI-TOF) genotyping of 10 SNPs centered on these nine candidate genes was confirmed. GO enrichment analysis revealed that genes within 1 Mb of the associated SNPs are significantly enriched in pathways involved in lymphocyte activation, in response to hydrogen peroxide, Salmonella infection, and other metabolic processes. This study explores the gene regulatory pathways influencing pH traits in goose meat and provides molecular markers for enhancing meat quality. These findings are expected to facilitate the advancement of molecular breeding programs in geese.

## Simple Summary

The pH is an important indicator in evaluating the quality of goose meat. SNP genotyping combined with genome-wide association studies (GWAS) is a common method for gene mining related to meat quality traits and has been widely applied in animal breeding research. However, gene mining studies focusing on goose meat quality traits have not yet been conducted. To explore the genetic characteristics of pH traits in goose meat, it is essential to study the SNPs and key candidate genes associated with the pH traits of Sichuan White Goose. In this study, we analyzed 203 male Sichuan White Geese. The results showed that 30 SNPs were associated with the pH traits of Sichuan White Goose meat, and 14 key candidate genes were identified. Our research provides a new perspective for improving goose meat quality and advancing molecular breeding practices.

## 1. Introduction

Goose meat, though only 2% of global poultry production, is crucial for dietary diversity and nutritional security due to its high protein content with an ideal amino acid composition, low fat and cholesterol levels, and high unsaturated fatty acids; endorsed by the World Health Organization, these qualities make goose meat a vital source of nutritious food and a key contributor to global food security and optimal nutrition [1]. The pH is a widely used parameter to assess the quality traits of goose meat. It serves as a critical indicator, reflecting the extent of glycolysis in the muscle post slaughter. Following slaughter, mitochondrial energy production and muscle metabolism cease, leading to enhanced glycolysis. The resulting pyruvic acid (CH3COCOOH) cannot enter the TCA cycle, causing a significant accumulation of lactic acid and a subsequent drop in meat pH [2]. The rate at which pH declines is closely linked to meat quality. The normal pH of chicken meat ranges between 5.8 and 6.2; values below 5.7 are classified as “PSE-like meat”, while those above 6.0 are classified as “DFD meat” [3]. As economic levels rise and consumer preferences shift, the demand for high-quality goose meat is growing, with an emphasis on taste and flavor, prompting the poultry industry to explore ways to reduce PSE meat [4].

Sichuan White goose has the characteristics of fast growth rate, high meat yield, and fresh and tender meat quality. Therefore, it is particularly important to find the genes that determine meat quality. Nevertheless, research on the quality of poultry meat has hitherto concentrated on chickens and ducks. Many scholars have extensively carried out genome-wide association analysis on meat quality traits such as pH and meat color and flavor in chickens and ducks and have explored multiple SNPs and candidate genes related to meat quality [5,6,7]. Studies on goose meat quality indexes mainly focus on differences among breeds, feed types, slaughtering methods and processing technologies [8,9]. Research on the quality of goose meat is very limited. In other farm animals, it was found that meat quality traits were affected by multiple genes. Point mutations in the good purine receptor gene (*RYR1*), for example, cause pale, soft, oozing PSE meat.

A splice mutation in the Phk γ subunit (*PHKG1*) gene, which encodes for the phosphorylase gamma subunit, has been demonstrated to affect glycogen content in skeletal muscle in porcine, leading to an increase in the proportion of acidic meat [10,11]. Notably, non-synonymous substitution I199 V in the *PRKAG3* gene can alter glycolytic potential (GP), which then affects pork quality traits, including water holding capacity (drop loss %) and pH [12].

Genome-wide association studies (GWASs) have become pivotal in linking single nucleotide polymorphisms (SNPs) with functional genes, widely applied in poultry and livestock to identify economically significant traits [13]. Demonstrating high accuracy and efficiency, GWASs have identified numerous causal variants and genes across various farm animals, producing comprehensive association profiles that enhance breeding programs, leading to significant achievements in cattle [14], sheep [15], pigs [16], chickens [17], and geese [18], providing a theoretical basis for future molecular breeding and meat quality improvements. Additionally, GWASs have facilitated the rapid localization of quantitative trait loci (QTL) and the identification of candidate genes related to meat quality traits [19]. For instance, in bovine studies, a QTL on chromosome 14 affecting meat pH has been identified, encompassing differentially expressed genes such as *FABP*, *CAPN1*, and *CAST*, which play crucial roles in beef maturation and carcass quality [20]. Research has also highlighted the *SOX6* gene as critical for chicken breast muscle development, identifying a haplotype containing 16 SNPs [21], and the *ABCG2* gene has been implicated in modulating egg color [22,23]. Furthermore, significant loci linked to disease resistance have been identified, offering new insights into the genetic determinants of health [24]. Our laboratory’s recent advancements include identifying numerous genes associated with reproduction, egg quality, meat quality, and body composition using SNP-GWAS and PAV-GWAS methodologies [25,26,27].

Understanding the genetic mechanisms regulating goose meat pH remains incomplete, hindering progress in meat quality enhancement and molecular breeding. Previously, our research group completed a GWAS on the meat quality (including meat color, shear force, and fatty acids. as shown in Supplementary Table S5) of Sichuan white geese in order to further understand the relationship between the pH of goose meat and other factors, such as growth, body size, and slaughter shape, as well as to identify which genes potentially regulate the pH of goose meat and thus affect the meat quality of the goose. In this study, we analyzed 203 healthy male Sichuan white geese, slaughtered at 70 days of age. We evaluated the pH of the meat, along with growth traits, body measurements, and slaughter characteristics. Pearson correlation analysis was utilized to determine the relationships between these traits. Whole genome resequencing was performed on the Illumina HiSeq X Ten platform, and high-quality SNP sites were obtained using BWA (version 0.7.17) software and GATK (version 3.8) software analysis, and GWASs and significant SNP analysis was performed upstream and downstream of a 100 kb gene annotation to identify candidate genes. These findings are expected to substantially advance molecular breeding efforts and provide significant theoretical and commercial value.

## 2. Materials and Methods

The experimental protocols involving Sichuan white geese were conducted in accordance with the guidelines provided by the Animal Ethics Committee Chongqing Academy of Animal Sciences.

### 2.1. Experimental Animals

This study was conducted at the Anfu Waterfowl Breeding Base in Chongqing, China (latitude: 105.478° N, longitude: 29.343° E). All animal experiments were performed in accordance with the guidelines set forth by the Animal Health and Welfare Committee of the Chongqing Academy of Animal Sciences. The experimental subjects were male Sichuan white geese from the same hatch batch. The diet followed international NRC (v1994) [28] standards, and the geese were provided with routine immunizations and ad libitum access to food and water.

### 2.2. Trait Measurement

A total of 203 geese were weighed at 6 weeks (42 days), 8 weeks (56 days), and 10 weeks (70 days) to assess growth performance. Key parameters measured included initial body weight (IBW/g), final body weight (FBW/g), and carcass weight (CW/g). To better record and differentiate the weights of geese at 6, 8, and 10 weeks of age, in this study, the weight recorded at 6 weeks was defined as the initial body weight (IBW/g), weight at 8 weeks was defined as the final body weight (FBW/g) and body weight at 10 weeks was defined as slaughter weight (SW/g). Blood samples (7 mL) were collected from the wing vein using vacuum tubes containing the anticoagulant EDTA. After standing, the blood was centrifuged at 3000 rpm for 10 min to obtain serum, which was then stored at −20 °C for subsequent analysis.

To determine the pH of meat, the pH of 203 right pectoral muscle samples at three fixed points was measured 2 h after slaughter using an appropriately calibrated portable pH meter (LP115, Metall Zug Group, Zurich, Switzerland). The measurements were taken at a depth of 1 cm after the proper calibration of the instrument. Additional measurements included slaughter weight (SW/g), dressed percentage (DP/%), heart weight (HW/g), pectoral muscle weight (PMW/g), glandular stomach weight (GSW/g), gizzard weight (GW/g), liver weight (LW/g), leg muscles (unilateral) heavy (LMH/g), and abdominal fat weight (AFW/g). Body size traits measured at 10 weeks included fossil bone length (FBL/cm), body slope length (BSL/cm), pelvis width (PW/mm), breast depth (BD/mm), breast width (BW/mm), shank length (SL/mm), half-diving depth (HDD/cm), and neck length (NL/cm).

The measurement methods adhered to the NY/T 823-2004 standards [29,30] for poultry performance terminology and statistical methods. Performance indices were calculated as follows: (1) average daily weight gain (ADG/g·d^−1^) = total weight gain/trial days; (2) slaughter rate = slaughter weight/live weight before slaughter × 100%.

### 2.3. Pearson’s Correlation Analysis Among Traits

Data were organized using Excel 2010, and statistical analyses were conducted using SPSS software (IBM SPSS Statistics Version 27.0, IBM Corp., Armonk, NY, USA). All phenotypic values were preliminarily sorted in Excel 2010, and the extreme phenotypic values of each type (values outside the mean ± 3 times standard deviation range) were removed. Statistical analysis software SPSS27.0 was used. The basic statistics such as mean value, standard deviation, maximum value, minimum value, and the coefficient of variation of the measured data of 22 traits were analyzed. The formula for calculating the coefficient of variation is as follows:

$$CV\left(\%\right)=\frac{S}{\bar{x}}$$

where S is the standard deviation and $\bar{x}$ is the average value.

The Pearson correlation analysis between pH and each trait was performed using the correlation (bivariate) module in the analysis of SPSS software. The correlation coefficient calculation formula is as follows:

$$p=\frac{1}{n-1}\sum \left(\frac{xi-x}{Sx}\right)\left(\frac{yi-y}{Sy}\right)$$

In which p represents the correlation coefficient, n represents the sample size, and Sx and Sy represent the standard deviations of the random variables x and y. The value of the correlation coefficient p is between −1 and 1, indicating the gradual enhancement of the linear correlation.

### 2.4. SNP Detection

Genomic DNA was extracted from goose blood samples using the Ezup Column Blood Genomic DNA Extraction Kit (Sangon, Shanghai, China). For each sample, 200 uL of venous blood was taken and added to an anticoagulant, then placed in a 1.5 mL tube labeled with the corresponding sample number. If the volume was insufficient, buffer GA was added to make up the difference. RNase A, Proteinase K solution, and buffer were added sequentially, followed by a water bath and centrifugation. An amount of 200 uL of absolute ethanol was added, and the resulting solution and flocculent precipitate were sequentially added to the adsorption column CB3, centrifuged at 12,000 rpm (~134,000× *g*) for 30 s, and then buffer GD, rinse solution PW, drying, and other steps were performed. The eluted DNA was immediately used for quality testing. DNA concentration was measured using the Nanodrop 2000 nucleic acid concentration meter, ensuring that the OD260/280 ratio was between 1.8 and 1.89. The DNA concentration was accurately quantified using Qubit, ensuring that the concentration of the extracted DNA sample was greater than or equal to 15 ng/uL. A 1.0 uL DNA sample was subjected to 1% agarose gel electrophoresis (100 V, 45 min) to detect DNA degradation and integrity. The purity was checked with a spectrophotometer; the extracted samples met the requirements, with clear, single bands without smears, suitable for subsequent experiments.

Then, our team previously downloaded whole genome sequencing data with 10.89× coverage for 70 Sichuan white geese aged up to 215 days (https://www.ncbi.nlm.nih.gov/bioproject/PRJNA595357, accessed on 8 October 2022). Raw sequenced reads were filtered using NGS QC Toolkit (version 2.3.3) software to remove adapters and low-quality reads, resulting in clean reads. These filtered reads were aligned to the goose genome using BWA (version 0.7.17) software. Subsequently, SNP detection was performed using GATK (3.8.1) software, and the final set of SNP loci was obtained by filtering with Plink (version 1.90) software.

### 2.5. Genome-Wide Association Study (GWAS)

The GEMMA (version 0.98) software was used to construct a mixed linear model (MLM) to identify SNPs associated with muscle pH traits in geese. The model used is as follows:

(1) y=Mα+xβ+e

where *y* represents the phenotypic value of the trait, M represents the covariance matrix, α represents the vector of coefficients including the intercept, x represents the corresponding SNP, β represents the SNP effect, and e represents the random residuals.

Wald’s test statistics and Bonferroni’s correction were used to assess the significance of the association between SNPs and the phenotype and to adjust the association results. The “gap” package in R (V4.3.1) was used to draw Manhattan plots and quantile–quantile (Q-Q) plots to identify genes associated with muscle pH traits.

### 2.6. Gene Annotation

Using BEDTools (v2.30.0), significant SNP loci were mapped to the reference ge-nome of geese. Based on the physical locations of these SNPs from the genome-wide association study, genes located within 1 Mb upstream and downstream of each SNP in the goose reference genome were identified. Functional information and annotation analysis of the relevant genes were retrieved using the Metascape website. Finally, the candidate genes were further analyzed in conjunction with existing research reports.

## 3. Results

### 3.1. Descriptive Statistics of Traits

The descriptive statistical analysis, as detailed in Table 1, reveals key insights into the phenotypic characteristics of Sichuan white geese. The average meat pH is measured at 5.71, with a standard deviation of 0.12 and a coefficient of variation at 2.01%. Notably, both growth parameters and body measurements demonstrate coefficients of variation of under 20%, indicating a relative homogeneity within these traits. In contrast, slaughter traits such as pectoral muscle weight (PMW) and abdominal fat weight (AFW) display higher coefficients of variation, both surpassing 20%. This elevated variability underscores a substantial genetic improvement potential within these specific traits. Other traits, however, maintain the coefficients of variation below 10%, reflecting their stability across the population.

### 3.2. Pearson’s Correlation Analysis Among Traits in Sichuan White Geese

#### 3.2.1. Pearson’s Correlation Analysis Elucidating the Relationship Between Meat pH and Growth Traits in Sichuan White Geese

A bivariate Pearson correlation analysis was conducted on all phenotypic values using SPSS software to investigate the relationship between PH traits and growth, body size, and slaughter traits in 181 geese. Table 2 illustrates that the correlation analysis between meat pH and growth traits does not reveal any statistically significant associations (*p* > 0.05).

#### 3.2.2. Pearson’s Correlation Analysis Elucidating the Relationship Between Meat pH and Body Size Traits in Sichuan White Geese

As Table 3 indicates, there was no statistically significant correlation between pH and body size traits in goose meat (*p* > 0.05).

#### 3.2.3. Pearson’s Correlation Analysis Elucidating the Relationship Between Meat pH and Slaughter Traits in Sichuan White Geese

The results presented in Table 4 and Table 5 demonstrate that there is no statistically significant correlation between meat pH and other slaughter traits (*p* > 0.05).

### 3.3. Genome-Wide Association Analysis

**Figure 1:**
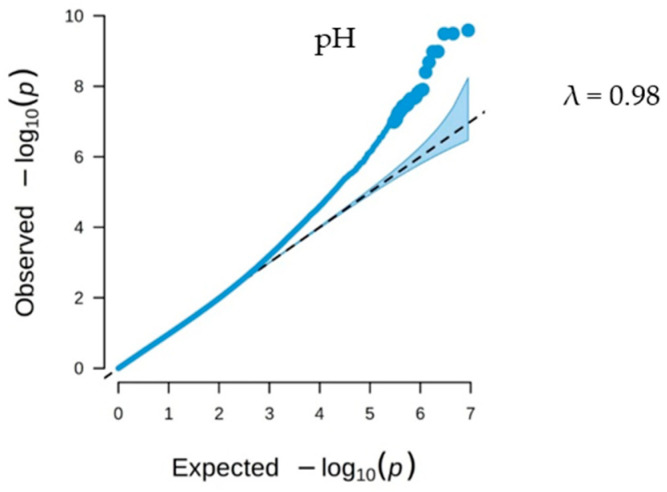
Shows the Q-Q plot for the genome-wide association analysis of meat pH in Sichuan white geese. The x-coordinate represents the negative logarithm of the expected *p*-value. The y-coordinate represents the negative logarithm of the actual observed *p*-value, and the diagonal represents the predicted line. The scatter in the lower left corner of the Q-Q plot indicates sites of low significance, and the near coincidence of the lines in the lower left corner indicates that the analysis model is reasonable. When the scatter is above the diagonal line, it indicates that these sites are significantly correlated with the traits.

It can be seen from Figure 1 in the Q-Q plot of SNPs associated with the pH of goose meat that most *p* values reside on the diagonal line, indicating that the observed and expected values are in good agreement. Additionally, the *p* values for those SNPs above the diagonal line are associated with the pH trait. A genome-wide association study (GWAS) was performed on meat pH using the mixed linear model (MLM) within the GEMMA (version 0.98) software framework. This analysis identified a total of 30 single nucleotide polymorphisms (SNPs) exhibiting significant and potential correlations with meat pH (Table 6). The SNP density in the goose genome within 1 Mb window size is shown in Supplementary Figure S1.

**Figure 2:**
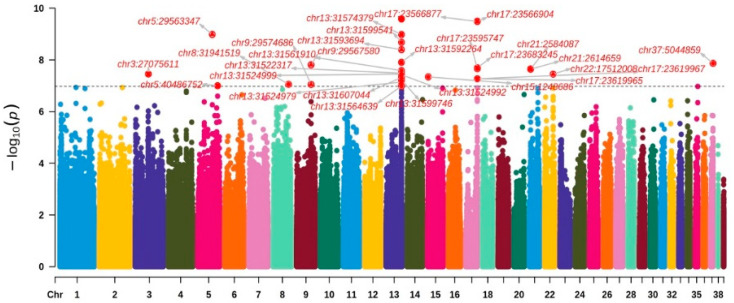
The Manhattan plot for the genome-wide association analysis of meat pH in Sichuan white geese. Note: In the Manhattan plot, the *X*-axis represents the chromosome position, the *Y*-axis represents the *p*-value size, and the dashed blue line represents the genomic significance threshold line.

The Manhattan plot shows that a significant number of SNPs across the genome level were detected in the pH phenotype data of Sichuan white goose meat, and 10 SNP loci were detected at the significance level (Figure 2). The 10 SNP loci identified as being significantly correlated with pH in goose meat underwent genotyping and annotation. Among the ten SNP loci found to be significantly associated, two were situated on chromosome 13 and positioned within the region (chr13: 31.52–31.61 Mb) adjacent to the *C19L2* gene. The remaining two were located on chromosome 17 and situated in the region (chr17: 23.57–23.68 Mb) close to the *RERGL* and *POL* genes. Additionally, another two were identified on chromosome 21, within the region (chr21: 2.58–2.61 Mb), near the *GK* and *AMFR* genes (Table 6).

### 3.4. Validating Association of SNPs with Meat pH

We used matrix-assisted laser desorption/ionized time-of-flight (MALDI-TOF) techniques to verify the genotypes of 203 Sichuan white geese. Ten SNPs were found to be significantly correlated with pH in goose meat [including (chr37:5044859, *p*-value = 0.031), (chr17:23683245, *p*-value = 0.041), (chr21:2614659, *p*-value = 0.009), (chr15:1248686, *p*-value = 0.016), (chr8:31941519, *p*-value = 0.029), (chr5:29563347 *p*-value = 0.002), (chr13:31599746, *p*-value = 0.016), (chr17:23566904, *p*-value = 0.037), (chr21:2584087, *p*-value = 0.019) and (chr13:31522317, *p*-value = 0.041)] (as shown in Table 7).

### 3.5. Gene Enrichment Analysis of Identified Significant SNPs

**Figure 3:**
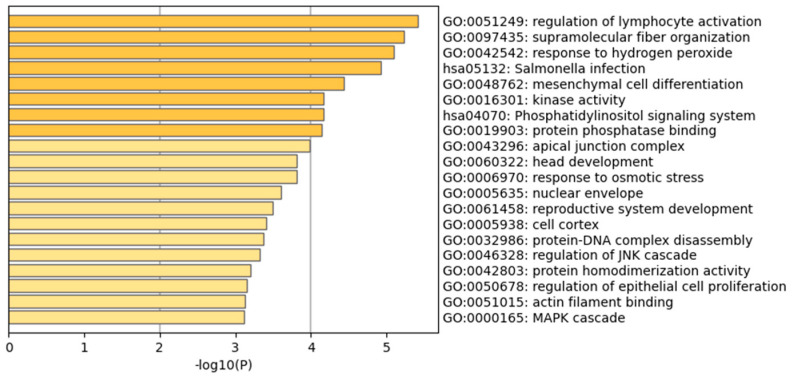
Functional analysis of genes located within 1 Mb of SNPs associated with muscle pH traits in geese. Supplementary Figure S1 shows the SNP density in the goose genome within the 1 Mb window size. Supplementary Figure S2 shows the KEGG analysis for the genes within 1 Mb in SNPs associated with goose meat quality traits.

In this study, the GO enrichment analysis of all SNPs related to pH traits showed that SNPs associated with pH traits of Sichuan white geese were mainly enriched in the regulation of lymphocyte activation (GO:0097435, 16/592, −LogP = −5.24) and in response to hydrogen peroxide (GO:0042542, 7/101, −LogP = −5.09), Salmonella infection (GO: hsa05132, 10/247, −LogP = −4.93), and other metabolic processes (Figure 3, Table 6 and Supplementary Tables S6–S8).

## 4. Discussion

Sichuan white geese are highly valued in China for their fine-textured, high-protein, and low-fat meat, which offers substantial economic benefits. A critical determinant of meat quality in these geese is muscle pH, which reflects the rate of glycogen glycolysis. The extent of pH decline influences key meat attributes such as meat color and water-holding capacity [31,32]. An elevated pH deepens meat color and enhances tenderness, whereas a lower pH diminishes water-holding capacity. Rapid post-slaughter pH reduction can inhibit protease activity, leading to decreased tenderness, pale meat, and reduced water-holding capacity. In this study, we systematically evaluated slaughter, growth, body size, and muscle pH characteristics in Sichuan white geese, finding an average muscle pH of 5.70. This value deviates from the 6.0 to 7.0 range reported by Koohmaraie et al. [33], likely due to differences in post-slaughter pH measurement timing. These findings highlight the importance of muscle pH in optimizing meat quality and provide empirical support for genetic improvement in geese.

To explore SNPs and candidate genes that influence PH in goose meat, we performed GWAS analysis on all geese. A genome-wide association study (GWAS) conducted on 203 male Sichuan white geese aged 70 days identified a total of 30 single nucleotide polymorphisms (SNPs) associated with meat pH. The Q-Q plot showed a good fit between observed and expected values, indicating no significant population stratification, so the reliability of the test results is demonstrated. The GWAS analysis identifies ten genomic regions that exhibit significant associations with meat pH traits, as indicated by single nucleotide polymorphisms (SNPs) located at chr37:5044859, chr17:23683245, chr21:2614659, chr15:1248686, chr8:31941519, chr5:29563347, chr13:31599746, chr17:23566904, chr21:2584087, and chr13:31522317. Validation using MALDI-TOF MS confirmed the correspondence between candidate SNPs and GWAS results, providing theoretical support for breeding programs aimed at improving meat quality and offering new directions for developing genetic markers.

To date, genome-wide association studies (GWASs) have been widely used in the molecular markers of quality in cattle, pigs, and chicken. Many other scholars and experts have carried out GWASs on the meat quality of cattle and pigs, and the results obtained are presented in the following section. Liu et al. conducted a genome-wide association study on meat quality traits of Beijing-You chickens and identified seven important SNPS and four candidate genes (*LCORL*, *LAP3*, *LDB2*, *TAPT1*) in the chromosome 4 region. Marin-Garzon and his colleagues used genome-wide techniques to detect genomic regions and candidate genes associated with the Nellore beef color trait. Similar genomic regions located on BTA chromosomes 2, 5, 6, and 18 were detected. The overlapping regions contained a total of 30 functionally annotated candidate genes involved in the regulation of muscle pH. Wang et al. genotyped 223 four-way hybrid pigs and identified a total of 64 SNPs for six meat quality traits in a genome-wide association study (GWAS) on pork quality traits, providing a large amount of new evidence for the involvement of candidate genes in different pork quality traits [34,35,36]. Based on whole genome resequencing, 203 Sichuan white geese were sequenced by 10× sequencing, and 10 SNPs loci with significant association levels were obtained by whole-genome association analysis with pH phenotype. Compared with most genome-wide association analysis studies on chips, resequencing has a comprehensive coverage of data sites, and the results are more accurate.

Gene function annotation identified nine candidate genes that were significantly associated with meat pH phenotypes, predominantly located on chromosomes 13 and 17, among these, the *C19L2* gene on chromosome 13 is involved in RNA metabolism and splicing, with high expression in testicular germ cells, although its direct relationship with meat pH remains unestablished [37,38]. Studies suggest that Leydig cells in the testes secrete hormones such as testosterone and growth hormone, which promote muscle protein synthesis, while cortisol facilitates protein degradation. These processes generate acidic or alkaline by-products, indirectly influencing meat pH [39,40,41]. The physiological functions and molecular mechanisms of the *C19L2* gene warrant further investigation.

Other identified genes, including *AMFR* (chr21:2584087), *GK* (chr21:2614659), *Pol* (chr17:23683245), and *WAC* (chr5:29563347), may indirectly affect meat pH through their roles in protein synthesis and metabolism [42,43,44,45,46,47,48,49]. *AMFR*, also known as *Gp78*, is an E3 ubiquitin ligase implicated in protein degradation. The Pol family of RNA polymerases plays a critical role in genetic information transfer, with Pol I involved in rRNA transcription and protein synthesis. *WAC*, with its WW and coiled coil domains, is integral to the ubiquitin–proteasome pathway, a key mechanism for protein degradation in cells. Notably, *AMFR* has also been linked to cancer metastasis, suggesting its potential as a biomarker for disease progression.

Additionally, meat pH, a direct indicator of tissue acidity or alkalinity, is crucial for assessing meat freshness, water-holding capacity, and texture. The disruption of the TCA cycle and gluconeogenesis in the animal body can alter meat pH [50,51]. The *GK* gene, encoding glycerol kinase, plays a pivotal role in lipid metabolism, and its disruption can lead to excessive lactic acid production, affecting meat pH [52,53,54]. The identified *GATA6*, *RERGL*, and *GMDS* genes, although not directly linked to goose meat pH, are key factors in various metabolic pathways and warrant further investigation to fully elucidate their roles in meat pH regulation and broader implications for animal breeding and human health.

The *GATA6*, *RERGL*, and *GMDS* genes identified in this study, although not directly linked to goose muscle pH in current bioinformatics databases, are pivotal in several key biological pathways. Further research is warranted to elucidate their potential roles in determining goose meat quality traits. Notably, these genes are integral to essential biological processes. *GATA6*, a member of the GATA transcription factor family, is expressed during early embryogenesis and later localizes to cells derived from the endoderm and mesoderm. Evidence suggests that *GATA6* functions as an oncogenic factor and is crucial for the development of the human digestive, endocrine, reproductive, and nervous systems [55,56]. *RERGL*, belonging to the Ras superfamily, mediates critical pathways for growth factors, cytokines, and extracellular signals, thereby regulating cell growth, differentiation, survival, and proliferation [57]. *GMDS* encodes GDP-mannose 4,6-dehydratase, an enzyme essential for converting GDP-mannose to GDP-4-keto-6-de-oxymannose, a key step in GDP-fucose synthesis. This enzyme plays a vital role in the N-glycosylation pathway, impacting protein folding, stability, activity, and intracellular transport [58,59,60,61,62]. Continued investigation into the functions and mechanisms of these genes is necessary to deepen our understanding of their roles in genetics and metabolism, with implications for accelerating animal breeding and providing insights into human cancer treatment models.

## 5. Conclusions

In this study, a genome-wide association study (GWAS) on pH traits in goose meat identified 10 significant SNPs, mainly located on chromosome 13 and 17, including candidate genes *C19L2*, *RERGL*, and *POL*. Notably, the *C19L2* gene could potentially be a key factor in regulating meat pH. The genetic markers identified offer new insights for the assisted selection of goose meat quality, providing valuable tools for breeding programs aimed at developing superior goose varieties.

## Figures and Tables

**Table 1:** Provides a comprehensive summary of the descriptive statistics for muscle pH, growth parameters, body measurements, and slaughter traits in Sichuan white geese.

Items	Mean	Number	STDV	Minimum	Maximum	CV(%)
pH	5.71	181	0.12	5.51	6.31	2.01
Growth Performance						
IBW/g	2095.00	203	265.53	1414.00	2863.00	12.67
FBW/g	2907.84	202	290.25	2120.00	3673.00	9.98
ADG/g·d^−1^	58.65	199	10.86	28.21	89.64	18.52
Body Size Traits						
FBL/cm	13.72	201	0.76	12.00	15.60	5.51
BSL/cm	28.04	198	1.14	24.80	30.60	4.08
PW/mm	72.86	201	3.64	64.41	82.39	4.99
BD/mm	114.24	200	7.07	93.99	133.60	6.19
BW/mm	122.56	201	6.94	105.51	142.50	5.67
SL/mm	121.33	199	4.49	107.94	133.83	3.70
HDD/cm	69.17	200	2.55	61.80	76.10	3.69
NL/cm	27.35	201	1.62	23.00	31.50	5.93
Slaughter Traits						
SW/g	3228.35	200	334.68	2369.00	4038.00	10.37
CW/g	2790.24	185	302.29	1970.00	3555.00	10.83
DP/%	87.28	183	2.67	80.42	93.92	3.06
HW/g	23.51	187	2.97	16.74	31.79	12.62
PMW/g	100.58	186	39.10	12.60	203.00	38.88
GSW/g	11.32	184	1.94	7.25	17.69	17.13
GW/g	105.05	184	14.71	69.10	146.10	14.00
LW/g	87.55	185	12.72	56.82	125.38	14.53
LMH/g	214.05	187	25.43	141.50	266.10	11.88
AFW/g	49.68	186	24.38	4.30	104.50	49.07

Note: All phenotypic values in Table 1 were collated to supplement the original data shown in Table S1, with missing NA and deviations (phenotypic values were above the mean ± SD by three times) removed (as shown in Supplementary Table S2). IBW: initial weight at 6 weeks of age; FBW: final weight at 8 weeks of age; ADG: average daily gain; CW: carcass weight intake; SW: slaughter weight; DP: dressed percentage; FBL: fossil bone length; BSL: body slope length; PW: pelvis width; BD: breast depth; BW: breast width; SL: shank length; HDD: half-diving depth; NL: neck length; HW: heart weight; PMW: pectoral muscle weight; GSW: glandular stomach weight; GW: gizzard weight; LW: liver weight; LMH: leg muscles (unilateral) heavy; AFW: abdominal fat weight.

**Table 2:** Correlation coefficient between meat pH and growth traits of Sichuan white geese.

Items	pH	IBW	FBW	ADG
pH	1			
IBW	−0.029	1		
FBW	−0.062	0.176 *	1	
ADG	0.035	−0.030	0.172 *	1

Note: Pearson correlation analysis of pH and growth traits in goose meat. (*n* = 181). IBW: initial weight at 6 weeks of age; FBW: final weight at 8 weeks of age; ADG: average daily gain. * indicates significant correlation among the phenotypic traits (*p* < 0.05), Detailed correlation analysis between pH value and other index data is shown in Supplementary Tables S3 and S4.

**Table 3:** Correlation coefficient between meat pH and body size traits of Sichuan white geese.

Items	PH	FBL	BSL	PW	BD	BW	SL	HDD	NL
PH	1								
FBL	−0.098	1							
BSL	0.007	0.326 **	1						
PW	−0.015	0.382 **	0.118	1					
BD	−0.050	0.215 **	0.165 *	0.044	1				
BW	0.077	0.475 **	0.290 **	0.325 **	0.235 **	1			
SL	0.057	0.551 **	0.312 **	0.310 **	0.227 **	0.469 **	1		
HDD	0.039	0.526 **	0.257 **	0.362 **	0.067	0.359 **	0.568 **	1	
NL	0.022	0.392 **	0.174 *	0.184 **	0.261 **	0.327 **	0.397 **	0.562 **	1

Note: Pearson correlation analysis of pH and body size traits in goose meat. (*n* = 181). FBL: fossil bone length; BSL: body slope length; PW: pelvis width; BD: Breast depth; BW: breast width; SL: shank length; HDD: half-diving depth; NL: neck length. * indicates significant correlation among the phenotypic traits (*p* < 0.05), ** indicates significant correlation among the phenotypic traits (*p* < 0.01), which is the same as below.

**Table 4:** Correlation coefficient between meat pH and slaughter traits of Sichuan white geese.

Items	pH	SW	CW	DP	HW	PMW
pH	1					
SW	−0.018	1				
CW	0.026	−0.003	1			
DP	−0.064	0.052	0.096	1		
HW	0.017	0.136	−0.056	0.088	1	
PMW	0.028	−0.025	0.232 **	−0.061	−0.044	1

Note: Pearson correlation analysis of pH and slaughter traits in goose meat. (*n* = 181). LW: liver weight; LMH: leg muscles (unilateral) heavy; SW: slaughter weight; DP: dressed percentage; CW: carcass weight. ** indicates significant correlation among the phenotypic traits (*p* < 0.01), which is the same as below.

**Table 5:** Correlation coefficient between meat pH and slaughter traits of Sichuan white geese.

Items	pH	GSW	GW	LW	LMH	AFW
pH	1					
GSW	0.015	1				
GW	0.008	0.276 **	1			
LW	0.095	0.007	0.000	1		
LMH	0.101	−0.073	0.073	0.357 **	1	
AFW	−0.067	−0.111	−0.042	0.029	−0.002	1

Note: Pearson correlation analysis of pH and slaughter traits in goose meat. (*n* = 181). HW: heart weight; PMW: pectoral muscle weight; GSW: glandular stomach weight; GW: gizzard weight; AFW: abdominal fat weight. ** indicates significant correlation among the phenotypic traits (*p* < 0.01), which is the same as below.

**Table 6:** Details the single nucleotide polymorphisms (SNPs) that are significantly associated with breast muscle quality traits.

SNP	Position (bp)	Allele1	*p*-Value	Gene
chr13:31574342	31574342	G/T	2.59 × 10^−10^	*C19L2*
chr17:23566904	23566904	A/G	3.2 × 10^−10^	*RERGL*
chr17:23566877	23566877	C/T	3.25 × 10^−10^	*RERGL*
chr5:29563347	29563347	G/T	1.04 × 10^−9^	*WAC*
chr13:31574379	31574379	T/C	1.04 × 10^−9^	*C19L2*
chr13:31599541	31599541	A/C	2.09 × 10^−9^	*C19L2*
chr13:31593694	31593694	A/G	4.01 × 10^−9^	*C19L2*
chr13:31592264	31592264	G/A	1.24 × 10^−8^	*C19L2*
chr37:5044859	5044859	T/C	1.36 × 10^−8^	*RHG23*
chr9:29567580	29567580	A/G	1.55 × 10^−8^	*ABLM1*
chr17:23683245	23683245	G/A	2.02 × 10^−8^	*POL*
chr17:23595747	23595747	C/A	2.19 × 10^−8^	*GATA6*
chr21:2584087	2584087	T/C	2.23 × 10^−8^	*AMFR*
chr21:2614659	2614659	G/A	2.29 × 10^−8^	*GK*
chr13:31561910	31561910	G/T	2.59 × 10^−8^	*C19L2*
chr13:31522317	31522317	G/A	3.27 × 10^−8^	*C19L2*
chr3:27075611	27075611	T/G	3.51 × 10^−8^	*RTJK*
chr13:31524999	31524999	A/G	3.56 × 10^−8^	*C19L2*
chr22:17512008	17512008	A/G	3.56 × 10^−8^	*SLAIL*
chr13:31524992	31524992	A/G	3.75 × 10^−8^	*C19L2*
chr15:1248686	1248686	T/C	4.55 × 10^−8^	*ZN484*
chr13:31607044	31607044	G/T	4.76 × 10^−8^	*C19L2*
chr17:23619965	23619965	G/C	5.39 × 10^−8^	*POL*
chr17:23619967	23619967	A/T	5.39 × 10^−8^	*POL*
chr13:31599746	31599746	T/C	5.9 × 10^−8^	*C19L2*
chr13:31524979	31524979	G/A	7.21 × 10^−8^	*C19L2*
chr8:31941519	31941519	A/T	8.78 × 10^−8^	*GMDS*
chr9:29574686	29574686	T/C	8.9 × 10^−8^	*ABLM1*
chr5:40486752	40486752	G/A	9.99 × 10^−8^	*CRFR2*
chr13:31564639	31564639	T/G	1.04 × 10^−7^	*C19L2*

Note: The table shows the 30 detected pH-associated SNP sites. Only the SNP point mutation sites are given. The thresholds for detecting the significant and potentially significant associations of SNPs were 4.94 × 10^−9^ and 1 × 10^−7^, respectively. *C19L2*: CWF19-like protein 2; *RERGL*: Ras-related and estrogen-regulated growth inhibitor-like protein; *WAC*: WW domain-containing adapter protein with a coiled coil; *RHG23*: Rho GTPase-activating protein 23; *ABLM1*: Actin-binding LIM protein 1 ;*POL*: Pol polyprotein; *GATA6*: Transcription factor GATA-6; *AMFR*: E3 ubiquitin–protein ligase AMFR; *GK*: Phosphoglycerate kinase; *RTJK*: RNA-directed DNA polymerase from mobile element jockey; *SLAIL*: motif-containing protein-like SLAIN; *ZN484*: Zinc finger protein 484 ; *GMDS*: GDP-mannose 4,6 dehydratase; *CRFR2*: Corticotropin-releasing factor receptor 2.

**Table 7:** The genotypes of SNP loci associated with muscle pH in geese.

SNPs	Genotype	*Gene*	Mean ± SD	Frequency	*p* Value
chr37:5044859	CC	*RHG23*	5.68 ± 0.07 ^a^	0.88	0.031
	CT		5.62 ± 0.02 ^b^	0.12	
chr17:23683245	AA	*POL*	5.68 ± 0.07 ^a^	0.91	0.041
	AG		5.71 ± 0.06 ^b^	0.09	
chr21:2614659	AA	*GK*	5.67 ± 0.08 ^a^	0.82	0.009
	AG		5.71 ± 0.04 ^b^	0.18	
chr15:1248686	CC	*ZN484*	5.67 ± 0.06 ^a^	0.74	0.016
	TC		5.72 ± 0.08 ^b^	0.26	
chr8:31941519	AA	*GMDS*	5.72 ± 0.05 ^a^	0.04	0.029
	AT		5.69 ± 0.07 ^b^	0.05	
	TT		5.68 ± 0.07 ^c^	0.91	
chr5:29563347	GT	*WAC*	5.71 ± 0.04 ^a^	0.11	0.002
	TT		5.68 ± 0.07 ^b^	0.89	
chr13:31599746	CC	*C19L2*	5.68 ± 0.07 ^a^	0.52	0.016
	TC		5.68 ± 0.05 ^a^	0.33	
	TT		5.62 ± 0.14 ^b^	0.15	
chr17:23566904	AA	*RERGL*	5.71 ± 0.06 ^ab^	0.15	0.037
	AG		5.67 ± 0.06 ^a^	0.42	
	GG		5.69 ± 0.08 ^b^	0.43	
chr21:2584087	CC	*AMFR*	5.67 ± 0.08 ^a^	0.81	0.019
	TC		5.71 ± 0.04 ^b^	0.19	
chr13:31522317	AA	*C19L2*	5.68 ± 0.06 ^a^	0.84	0.041
	GA		5.71 ± 0.09 ^b^	0.16	

Note: The peer data shoulder label does not contain the same lowercase letters indicating significant differences (*p* < 0.05), and contains the same lowercase letters or no letters indicating no significant differences (*p* > 0.05); The following table is the same.

## Data Availability

Data are contained within the article.

## Supplementary Materials

The following supporting information can be downloaded at: https://www.mdpi.com/article/10.3390/biology13110865/s1. Table S1: The descriptive statistics of pH value, growth parameters, body size measurement and slaughter traits of Sichuan white geese were reviewed. Table S2: Comparison of pH, growth, body size and slaughter traits data between Table 1 and Supplementary Table S1. Table S3: Correlation analysis between PH and growth, body size and slaughter traits. Table S4: Correlation analysis between PH and growth, body size and slaughter traits. Table S6: GO enrichment analysis of Sichuan White geese. Table S7: GO enrichment analysis of the Sichuan White Geese (continuation of the table). Table S8: GO enrichment analysis of the Sichuan White Geese (continuation of the table). Figures S1,S2 and Table S5 can be downloaded at the following link: https://doi.org/10.3390/ani13132089 (accessed on 20 August 2024). Figure S1: The SNP density in the goose genome within 1 Mb window size. References can be found on Table S5: Descriptive statistics were calculated for the meat quality traits of male Sichuan white geese at 70 days of age. References can be found on Figure S2: The functional analysis for the genes within 1 Mb within SNPs associated with goose meat quality traits.

## References

[1] Kozák J. (2021). Goose production and goose products. World’s Poult. Sci. J..

[2] Matarneh S.K., Scheffler T.L., Gerrard D.E. (2023). The conversion of muscle to meat. Lawrie’s Meat Science.

[3] Dang K., Farooq HM U., Gao Y., Deng X., Qian A. (2023). The role of 5′-adenosine monophosphate-activated protein kinase (AMPK) in skeletal muscle atrophy. Biocell.

[4] Barbut S., Sosnicki A.A., Lonergan S.M., Knapp T., Ciobanu D.C., Gatcliffe L.J., Huff-Lonergan E., Wilson E.W. (2008). Progress in reducing the pale, soft and exudative (PSE) problem in pork and poultry meat. Meat Sci..

[5] Guo Q., Huang Z., Bi Y., Chen G., Chang G. (2022). Genome-wide association study of potential meat quality trait loci in ducks. Genes.

[6] Zhang F., Zhu F., Yang F.X., Hao J.P., Hou Z.C. (2022). Genomic selection for meat quality traits in Pekin duck. Animal Genetics. Anim. Genet..

[7] Sun Y., Zhao G., Liu R., Zheng M., Hu Y., Wu D., Zhang L., Li P., Wen J. (2013). The identification of 14 new genes for meat quality traits in chicken using a genome-wide association study. BMC Genom..

[8] Yan X., Xu Y., Zhen Z., Li J., Zheng H., Li S., Ye P. (2023). Slaughter performance of the main goose breeds raised commercially in China and nutritional value of the meats of the goose breeds: A systematic review. J. Sci. Food Agric..

[9] Zhang Y., Qi S., Fan S., Jin Z., Bao Q., Zhang Y., Chen G. (2024). Comparison of growth performance, meat quality, and blood biochemical indexes of Yangzhou goose under different feeding patterns. Poult. Sci..

[10] Marini S.J., Vanzetti L.S., Borelli V.S., Villareal A.O., Denegri G.D., Cottura G.A., Franco R. (2014). RYR1 gene variability and effect on meat pH in Argentinean hybrids swines. InVet.

[11] Ma J., Yang J., Zhou L., Ren J., Liu X., Zhang H., Huang L. (2014). splice mutation in the PHKG1 gene causes high glycogen content and low meat quality in pig skeletal muscle. PLoS Genet..

[12] Warner R.D., Greenwood P.L., Pethick D.W., Ferguson D.M. (2010). Genetic and environmental effects on meat quality. Meat Sci..

[13] Hoa V.B., Seol K.H., Seo H.W., Seong P.N., Kang S.M., Kim Y.S., Moon S.S., Kim J.H., Cho S.H. (2021). Meat quality characteristics of pork bellies in relation to fat level. Anim. Biosci..

[14] Sermyagin A.A., Bykova O.A., Loretts O.G., Kostyunina O.V., Zinovieva N.A. (2020). Genomic variability assess for breeding traits in holsteinizated Russian Black-and-White cattle using GWAS analysis and ROH patterns. Agric. Biol..

[15] Gebreselassie G., Berihulay H., Jiang L., Ma Y. (2020). Review on genomic regions and candidate genes associated with economically important production and reproduction traits in sheep (*Ovies aries*). Animals.

[16] Zhang Y., Zhang J., Gong H., Cui L., Zhang W., Ma J., Chen C., Ai H., Xiao S., Huang L. (2019). Genetic correlation of fatty acid composition with growth, carcass, fat deposition and meat quality traits based on GWAS data in six pig populations. Meat Sci..

[17] Zhang H., Shen L.-Y., Xu Z.-C., Kramer L.M., Yu J.-Q., Zhang X.-Y., Na W., Yang L.-L., Cao Z.-P., Luan P. (2020). Haplotype-based genome-wide association studies for carcass and growth traits in chicken. Poult. Sci..

[18] Gao G., Gao D., Zhao X., Xu S., Zhang K., Wu R., Yin C., Li J., Xie Y., Hu S. (2021). Genome-Wide association study-based identification of SNPs and haplotypes associated with goose reproductive performance and egg quality. Front. Genet..

[19] Munyaneza J.P., Ediriweera T.K., Kim M., Cho E., Jang A., Choo H., Lee J.H. (2022). Genome-wide association studies of meat quality traits in chickens: A review. Korean J. Agric. Sci..

[20] Sun X., Wu X., Fan Y., Mao Y., Ji D., Huang B., Yang Z. (2018). Effects of polymorphisms in CAPN1 and CAST genes on meat tenderness of Chinese Simmental cattle. Arch. Anim. Breed..

[21] Tan X., Liu R., Zhao D., He Z., Li W., Zheng M., Li Q., Wang Q., Liu D., Feng F. (2024). Large-scale genomic and transcriptomic analyses elucidate the genetic basis of high meat yield in chickens. J. Adv. Res..

[22] Chen L., Gu X., Huang X., Liu R., Li J., Hu Y., Li G., Zeng T., Tian Y., Hu X. (2020). Two cis-regulatory SNPs upstream of ABCG2 synergistically cause the blue eggshell phenotype in the duck. PLoS Genet..

[23] Liu H., Hu J., Guo Z., Fan W., Xu Y., Liang S., Liu D., Zhang Y., Xie M., Tang J. (2021). A single nucleotide polymorphism variant located in the cis-regulatory region of the ABCG2 gene is associated with mallard egg colour. Mol. Ecol..

[24] Walker L.R., Engle T.B., Vu H., Tosky E.R., Nonneman D.J., Smith T.P.L., Borza T., Burkey T.E., Plastow G.S., Kachman S.D. (2018). Synaptogyrin-2 influences replication of Porcine circovirus 2. PLoS Genet..

[25] Gao G.L., Zhang K.-S., Zhao X.-Z., Xu G.Y., Xie Y.H., Zhou L., Zhang C.-L., Wang Q.-G. (2023). Identification of molecular markers associated with goose egg quality through genome-wide association analysis. Sci. Agric. Sin..

[26] Gao G., Zhang K., Huang P., Zhao X., Li Q., Xie Y., Yin C., Li J., Wang Z., Zhong H. (2023). Identification of snps associated with goose meat quality traits using a genome-wide association study approach. Animals.

[27] Gao G., Zhang H., Ni J., Zhao X., Zhang K., Wang J., Kong X., Wang Q. (2023). Insights into genetic diversity and phenotypic variations in domestic geese through comprehensive population and pan-genome analysis. J. Anim. Sci. Biotechnol..

[28] National Research Council (1994). Nutrient Requirements of Poultry: Ninth Revised Edition, 1994.

[29] Tyasi T.L., Qin N., Jing Y., Mu F., Zhu H.Y., Liu D., Xu R. (2017). Assessment of relationship between body weight and body measurement traits of indigenous Chinese Dagu chickens using path analysis. Indian J. Anim. Res..

[30] NY/T 823-2004. https://www.chinesestandard.net/PDF/BOOK.aspx/NYT823-2004.

[31] Yuan Y., Deng W., Jin Y., Li W., Li S. (2020). Research progress of pork quality evaluation index and influencing factors. Heilongjiang Anim. Husb. Vet. Sci..

[32] Wang H.P. (2011). Sensory evaluation of Longissimus dorsi muscle of pigs: Relationship between postmortem meat quality traits and muscle fiber characteristics. Meat Res..

[33] Koohmaraie M., Kent M.P., Shackelford S.D., Veiseth E., Wheeler T.L. (2002). Meat Tenderness and Muscle Growth: Is There Any Relationship?. Meat Sci..

[34] Marín-Garzón N., Magalhães A., Mota L., Fonseca L., Chardulo L., Albuquerque L. (2021). Genome-wide association study identified genomic regions and putative candidate genes affecting meat color traits in Nellore cattle. Meat Sci..

[35] Wang H., Wang X., Li M., Sun H., Chen Q., Yan D., Lu S. (2023). Genome-wide association study reveals genetic loci and candidate genes for meat quality traits in a four-way crossbred pig population. Front. Genet..

[36] Liu R., Sun Y., Zhao G., Wang F., Wu D., Zheng M., Wen J. (2013). Genome-wide association study identifies loci and candidate genes for body composition and meat quality traits in Beijing-You chickens. PLoS ONE.

[37] Kraemer W.J., Ratamess N.A., Hymer W.C., Nindl B.C., Fragala M.S. (2020). Growth hormone (s), testosterone, insulin-like growth factors, and cortisol: Roles and integration for cellular development and growth with exercise. Front. Endocrinol..

[38] Wang S., Cai Y., Li T., Wang Y., Bao Z., Wang R., Qin J., Wang Z., Liu Y., Liu Z. (2024). CWF19L2 is Essential for Male Fertility and Spermatogenesis by Regulating Alternative Splicing. Adv. Sci..

[39] Xiong Y.L. (2018). Muscle proteins. Proteins in Food Processing.

[40] Sánchez-Velázquez J., Peña-Herrejón G.A., Aguirre-Becerra H. (2024). Fish Responses to Alternative Feeding Ingredients under Abiotic Chronic Stress. Animals.

[41] Ge R.S., Li X., Wang Y. (2021). Leydig cell and spermatogenesis. Mol. Mech. Spermatogenesis.

[42] Joshi V., Upadhyay A., Kumar A., Mishra A. (2017). Gp78 E3 ubiquitin ligase: Essential functions and contributions in proteostasis. Front. Cell. Neurosci..

[43] Chen J., Chen H.-H., Luo L.-H., Kang R.-H., Liang S.-J., Zhu Q.-Y., Lu H.-Z., Liu X., Chen Y., Feng Y. (2023). Paired comparison of molecular transmission networks based on HIV-1 pol gene DNA or RNA sequences. China Trop. Med..

[44] Khatter H., Vorländer M.K., Müller C.W. (2017). RNA polymerase I and III: Similar yet unique. Curr. Opin. Struct. Biol..

[45] Dieci G., Fiorino G., Castelnuovo M., Teichmann M., Pagano A. (2007). The expanding RNA polymerase III transcriptome. TRENDS Genet..

[46] Rudolph H.C., Stafford A.M., Hwang H.-E., Kim C.-H., Prokop J.W., Vogt D. (2023). Structure-Function of the Human WAC Protein in GABAergic Neurons: Towards an Understanding of Autosomal Dominant DeSanto–Shinawi Syndrome. Biology.

[47] Clark C.R. (2018). Characterization of TM9SF2 and WAC as Novel Colorectal Cancer Driver Genes. Ph.D. Thesis.

[48] Zhang Y., Wang X., Chen G., Lu Y., Chen Q. (2024). Autocrine motility factor receptor promotes the malignancy of glioblastoma by regulating cell migration and invasion. Neurol. Res..

[49] Onishi Y., Haga A., Raz A. (2002). Autocrine Motility Factor and Its Receptor as Regulators of Metastasis. Cancer Metastasis—Related Genes.

[50] Kamel K.S., Oh M.S., Halperin M.L. (2020). L-lactic acidosis: Pathophysiology, classification, and causes; emphasis on biochemical and metabolic basis. Kidney Int..

[51] Hood V.L., Tannen R.L. (1998). Protection of acid–base balance by pH regulation of acid production. N. Engl. J. Med..

[52] Chen Y., Jiang H., Zhan Z., Lu J., Gu T., Yu P., Liang W., Zhang X., Liu S., Bi H. (2023). Restoration of lipid homeostasis between TG and PE by the LXRα-ATGL/EPT1 axis ameliorates hepatosteatosis. Cell Death Dis..

[53] Wan L., Zeng H., Peng L., Wen S., Liu C., Bao S., An Q., Huang J., Liu Z. (2024). Induction and mechanism of EGCG on the beigeing of white adipose tissue in GK rats with high-fat diet. J. Tea Sci..

[54] Thompson B., Satin L.S. (2021). Beta-cell ion channels and their role in regulating insulin secretion. Compr. Physiol..

[55] Viger R.S., Guittot S.M., Anttonen M., Wilson D.B., Heikinheimo M. (2008). Role of the GATA family of transcription factors in endocrine development, function, and disease. Mol. Endocrinol..

[56] Koutsourakis M., Langeveld A., Patient R., Beddington R., Grosveld F. (1999). The transcription factor GATA6 is essential for early extraembryonic development. Development.

[57] Yang R., Chen B., Pfütze K., Buch S., Steinke V., Holinski-Feder E., Stöcker S., von Schönfels W., Becker T., Schackert H.K. (2014). Genome-wide analysis associates familial colorectal cancer with increases in copy number variations and a rare structural variation at 12p12. 3. Carcinogenesis.

[58] Liu H.Y., Zhang C.J. (2017). Identification of differentially expressed genes and their upstream regulators in colorectal cancer. Cancer Gene Ther..

[59] Nakayama K., Moriwaki K., Imai T., Shinzaki S., Kamada Y., Murata K., Miyoshi E. (2013). Mutation of GDP-mannose-4, 6-dehydratase in colorectal cancer metastasis. PLoS ONE.

[60] Wang W., Zhang F., Wen Y., Hu Y., Yuan Y., Wei M., Zhou Y. (2019). Cell-free enzymatic synthesis of GDP-L-fucose from mannose. AMB Express.

[61] Moriwaki K., Shinzaki S., Miyoshi E. (2011). GDP-mannose-4, 6-dehydratase (GMDS) deficiency renders colon cancer cells resistant to tumor necrosis factor-related apoptosis-inducing ligand (TRAIL) receptor-and CD95-mediated apoptosis by inhibiting complex II formation. J. Biol. Chem..

[62] Yarema K.J. (2005). Mammalian Glycosylation: An Overview of Carbohydrate Biosynthesis. Handbook of Carbohydrate Engineering.

