# Supplementary material for: Identification of Single Nucleotide Polymorphisms Through Genome-Wide Association Studies of pH Traits in Goose Meat
Source: Biology (Basel). 2024 Oct 24;13(11):865. doi: 10.3390/biology13110865 (PMC11592244; doi:10.3390/biology13110865)
Supplement: Supplementary file 1 [file biology-13-00865-s001.zip › biology-3192939-supplementary.pdf]

Supplemental Table S5 and supplemental Figures S1 and S2 have already been published in other articles, and this article uses them for ease of understanding by readers, editors, and experts.

Table S1. The descriptive statistics of pH value, growth parameters, body size measurement and slaughter traits of Sichuan white geese were reviewed.

Items	Mean	Number	STDV	Minimum	Maximum	CV(%)
pH	5.71	203	0.12	5.51	6.31	2.01
Growth Performance						
IBW/g	2095.00	203	265.53	1414.00	2863.00	12.67
FBW/g	2907.84	203	290.25	2120.00	3673.00	9.98
ADG/g·d^{-1}	58.06	203	11.79	17.86	89.64	20.30
Body Size Traits						
FBL/cm	13.72	203	0.76	12.00	15.60	5.51
BSL/cm	28.05	203	1.30	21.80	32.80	4.62
PW/mm	72.86	203	3.64	64.41	82.39	4.99
BD/mm	114.36	203	7.24	93.99	137.78	6.33
BW/mm	122.56	203	6.94	105.51	142.50	5.67
SL/mm	121.14	203	4.87	99.69	133.83	4.02
HDD/cm	69.21	203	2.61	61.80	77.20	3.77
NL/cm	27.35	203	1.62	23.00	31.50	5.93
Slaughter Traits						
SW/g	3223.03	203	342.24	2160.00	4038.00	10.62
CW/g	2790.24	203	302.29	1970.00	3555.00	10.83
DP/%	87.08	203	3.33	65.63	93.92	3.82
HW/g	23.51	203	2.97	16.74	31.79	12.62
PMW/g	101.41	203	40.60	12.60	255.20	40.04
GSW/g	11.35	203	2.21	1.50	19.76	19.44
GW/g	105.16	203	17.46	5.50	165.10	16.60
LW/g	88.01	203	13.40	56.82	130.59	15.22
LMH/g	214.05	203	25.43	141.50	266.10	11.88
AFW/g	50.10	203	24.97	4.30	127.50	49.84

Table S2. Comparison of pH, growth, body size and slaughter traits data between Table 1 and Supplement Table S1

Items	Supplementary Table S1 Number	Table 1 Number	Missing Value	deviating Value
pH	203	181	22	0
Growth Performance				
IBW/g	203	203	0	0
FBW/g	203	202	1	0
ADG/g·d^{-1}	203	199	1	3
Body Size Traits				
FBL/cm	203	201	2	0
BSL/cm	203	198	2	3
PW/mm	203	201	2	0
BD/mm	203	200	2	1
BW/mm	203	201	2	0
SL/mm	203	199	2	2

HDD/cm	203	200	2	1
NL/cm	203	201	2	0
Slaughter Traits				
SW/g	203	200	2	1
CW/g	203	185	18	0
DP/%	203	183	18	2
HW/g	203	187	16	0
PMW/g	203	186	16	1
GSW/g	203	184	16	3
GW/g	203	184	16	3
LW/g	203	185	16	2
LMH/g	203	187	16	0
AFW/g	203	186	16	1

Note: deviations (phenotypic values beyond the mean ± 3 times SD).

Table S3 Correlation analysis between PH and growth, body size and slaughter traits

**correlation**										
		PH	IBW	FBW	ADG	FBL	BSL	PW	BD	BW
PH	Pearson correlation	1	-0.029	-0.062	0.035	-0.098	0.007	-0.015	-0.050	0.077
	P-Value		0.696	0.404	0.642	0.190	0.927	0.844	0.502	0.300
	Number	181	181	181	181	181	181	181	181	181
IBW	Pearson correlation	-0.029	1	0.176*	-0.030	0.082	0.012	0.024	0.085	-0.046
	P-Value	0.696		0.012	0.670	0.247	0.870	0.730	0.229	0.519
	Number	181	203	202	199	201	198	201	200	201
FBW	Pearson correlation	-0.062	0.176*	1	0.172*	0.308**	0.058	0.142*	0.220**	0.143*
	P-Value	0.404	0.012		0.015	0.000	0.415	0.045	0.002	0.042
	Number	181	202	202	199	201	198	201	200	201
ADG	Pearson correlation	0.035	-0.030	0.172*	1	0.270**	0.096	0.159*	0.043	0.173*
	P-Value	0.642	0.670	0.015		0.000	0.176	0.025	0.547	0.014
	Number	181	199	199	199	199	198	199	199	199
FBL	Pearson correlation	-0.098	0.082	0.308**	0.270**	1	0.326**	0.382**	0.215**	0.475**
	P-Value	0.190	0.247	0.000	0.000		0.000	0.000	0.002	0.000
	Number	181	201	201	199	201	198	201	200	201
BSL	Pearson correlation	0.007	0.012	0.058	0.096	0.326**	1	0.118	0.165*	0.290**
	P-Value	0.927	0.870	0.415	0.176	0.000		0.097	0.020	0.000
	Number	181	198	198	198	198	198	198	198	198
PW	Pearson correlation	-0.015	0.024	0.142*	0.159*	0.382**	0.118	1	0.044	0.325**
	P-Value	0.844	0.730	0.045	0.025	0.000	0.097		0.532	0.000
	Number	181	201	201	199	201	198	201	200	201

BD	Pearson correlation	-0.050	0.085	0.220**	0.043	0.215**	0.165*	0.044	1	0.235**
	P-Value	0.502	0.229	0.002	0.547	0.002	0.020	0.532		0.001
	Number	181	200	200	199	200	198	200	200	200
BW	Pearson correlation	0.077	-0.046	0.143*	0.173*	0.475**	0.290**	0.325**	0.235**	1
	P-Value	0.300	0.519	0.042	0.014	0.000	0.000	0.000	0.001	
	Number	181	201	201	199	201	198	201	200	201
*. $p < 0.05$ indicates significant correlation										
**. $P < 0.01$ indicates significant correlation										

Table S4 Correlation analysis between PH and growth, body size and slaughter traits

correlation															
		PH	SL	HDD	NL	SW	CW	DP	HW	PMW	GSW	GW	LW	LMH	AFW
PH	Pearson correlation	1	0.057	0.039	0.022	-0.018	0.026	-0.064	0.017	0.028	0.015	0.008	0.095	0.101	-0.067
	P-Value		0.447	0.606	0.768	0.808	0.724	0.394	0.823	0.710	0.843	0.913	0.203	0.178	0.369
	Number	181	181	181	181	181	181	181	181	181	181	181	181	181	181
SL	Pearson correlation	0.057	1	0.568**	0.397**	0.437**	0.003	-0.100	0.036	-0.003	-0.054	-0.082	-0.023	0.040	0.077
	P-Value	0.447		0.000	0.000	0.000	0.970	0.178	0.627	0.963	0.466	0.267	0.757	0.589	0.295
	Number	181	199	199	199	199	185	183	187	186	184	184	185	187	186
HDD	Pearson correlation	0.039	0.568**	1	0.562**	0.431**	-0.009	0.036	0.095	0.010	-0.036	-0.064	0.022	0.027	0.150*
	P-Value	0.606	0.000		0.000	0.000	0.904	0.626	0.198	0.888	0.632	0.388	0.766	0.716	0.041
	Number	181	199	200	200	200	185	183	187	186	184	184	185	187	186
NL	Pearson correlation	0.022	0.397**	0.562**	1	0.253**	-0.049	-0.010	0.079	-0.064	0.010	0.059	-0.004	0.061	0.080
	P-Value	0.768	0.000	0.000		0.000	0.509	0.893	0.285	0.384	0.894	0.428	0.952	0.407	0.276
	Number	181	199	200	201	200	185	183	187	186	184	184	185	187	186
SW	Pearson correlation	-0.018	0.437**	0.431**	0.253**	1	-0.003	0.052	0.136	-0.025	-0.012	0.022	0.043	0.102	0.049
	P-Value	0.808	0.000	0.000	0.000		0.971	0.485	0.064	0.730	0.867	0.765	0.558	0.163	0.505
	Number	181	199	200	200	200	185	183	187	186	184	184	185	187	186
CW	Pearson correlation	0.026	0.003	-0.009	-0.049	-0.003	1	0.096	-0.056	0.232**	0.212**	0.131	0.167*	0.074	0.139
	P-Value	0.724	0.970	0.904	0.509	0.971		0.195	0.450	0.002	0.004	0.077	0.023	0.314	0.058
	Number	181	185	185	185	185	185	183	185	185	184	184	185	185	185
DP	Pearson correlation	-0.064	-0.100	0.036	-0.010	0.052	0.096	1	0.088	-0.061	-0.096	0.015	0.165*	0.007	0.168*
	P-Value	0.394	0.178	0.626	0.893	0.485	0.195		0.237	0.413	0.194	0.839	0.026	0.925	0.023
	Number	181	183	183	183	183	183	183	183	183	183	183	183	183	183
HW	Pearson correlation	0.017	0.036	0.095	0.079	0.136	-0.056	0.088	1	-0.044	-0.038	0.016	0.366**	0.435**	0.041
	P-Value	0.823	0.627	0.198	0.285	0.064	0.450	0.237		0.547	0.609	0.830	0.000	0.000	0.578
	Number	181	187	187	187	187	185	183	187	186	184	184	185	187	186

PMW	Pearson correlation	0.028	-0.003	0.010	-0.064	-0.025	0.232**	-0.061	-0.044	1	-0.088	0.005	0.077	0.100	0.171*
	P-Value	0.710	0.963	0.888	0.384	0.730	0.002	0.413	0.547		0.235	0.951	0.297	0.175	0.020
	Number	181	186	186	186	186	185	183	186	186	184	184	185	186	186
GSW	Pearson correlation	0.015	-0.054	-0.036	0.010	-0.012	0.212**	-0.096	-0.038	-0.088	1	0.276**	0.007	-0.073	-0.111
	P-Value	0.843	0.466	0.632	0.894	0.867	0.004	0.194	0.609	0.235		0.000	0.920	0.327	0.133
	Number	181	184	184	184	184	184	183	184	184	184	184	184	184	184
GW	Pearson correlation	0.008	-0.082	-0.064	0.059	0.022	0.131	0.015	0.016	0.005	0.276**	1	0.000	0.073	-0.042
	P-Value	0.913	0.267	0.388	0.428	0.765	0.077	0.839	0.830	0.951	0.000		1.000	0.324	0.570
	Number	181	184	184	184	184	184	183	184	184	184	184	184	184	184
LW	Pearson correlation	0.095	-0.023	0.022	-0.004	0.043	0.167*	0.165*	0.366**	0.077	0.007	0.000	1	0.357**	0.029
	P-Value	0.203	0.757	0.766	0.952	0.558	0.023	0.026	0.000	0.297	0.920	1.000		0.000	0.691
	Number	181	185	185	185	185	185	183	185	185	184	184	185	185	185
LMH	Pearson correlation	0.101	0.040	0.027	0.061	0.102	0.074	0.007	0.435**	0.100	-0.073	0.073	0.357**	1	-0.002
	P-Value	0.178	0.589	0.716	0.407	0.163	0.314	0.925	0.000	0.175	0.327	0.324	0.000		0.978
	Number	181	187	187	187	187	185	183	187	186	184	184	185	187	186
AFW	Pearson correlation	-0.067	0.077	0.150*	0.080	0.049	0.139	0.168*	0.041	0.171*	-0.111	-0.042	0.029	-0.002	1
	P-Value	0.369	0.295	0.041	0.276	0.505	0.058	0.023	0.578	0.020	0.133	0.570	0.691	0.978	
	Number	181	186	186	186	186	185	183	186	186	184	184	185	186	186
*. $p < 0.05$ indicates significant correlation															
**. $P < 0.01$ indicates significant correlation															

Figure S1. The SNP density in the goose genome within 1Mb window size. References can be found on [26].

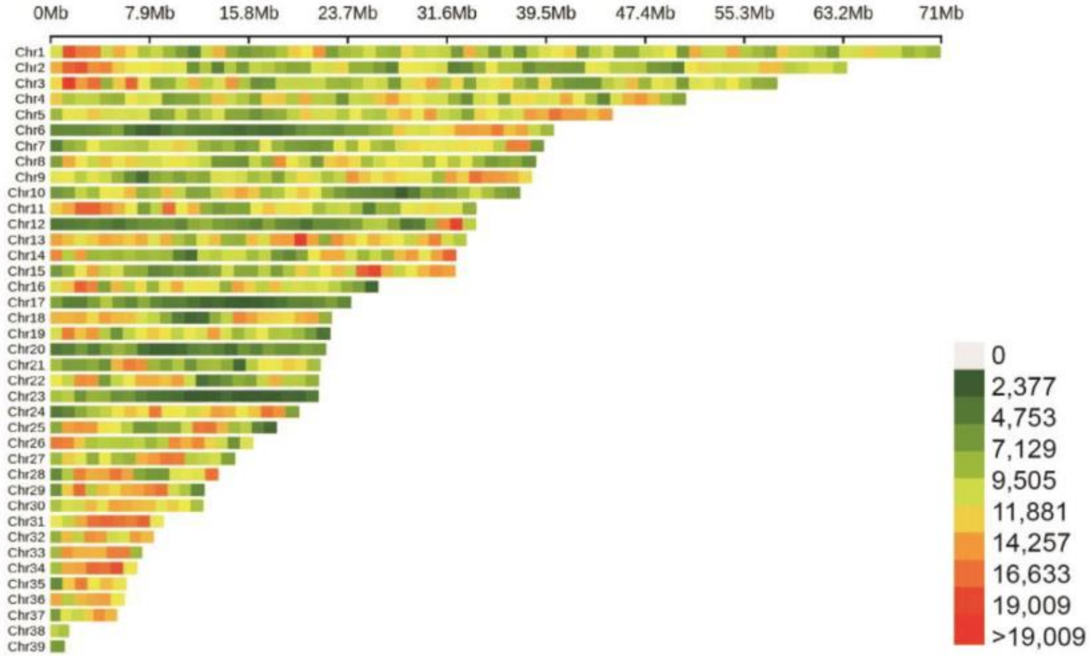

Table S5. Descriptive statistics were calculated for the meat quality traits of male Sichuan white geese at 70 days of age. References can be found on [26].

Traits	Number	Mean	STDV	Minimum	Maximum	CV (%)
CFC	199	9.92	1.89	4.69	15.52	0.19
CLR	194	13.13	3.01	4.66	22.36	0.23
L* (meat lightness)	205	23.6	3.65	15.53	37.29	0.15
a* (meat redness)	205	48.77	3.02	39.76	56.06	0.06
b* (meat yellowness)	205	19.5	2.32	13.05	30.8	0.12
SF (kgf)	197	3.81	0.83	1.66	5.82	0.22
SF (kgf)	197	3.81	0.83	1.66	5.82	0.22

Note: CFC: crude fat content; MCFD: moisture content of freeze dried; CLR: cooking loss rate; L*: meat lightness; a*: meat redness); b*: meat yellowness; SF (kgf): shear force.

Figure S2. The functional analysis for the genes within 1 Mb within SNPs associated with goose meat quality traits. [26]

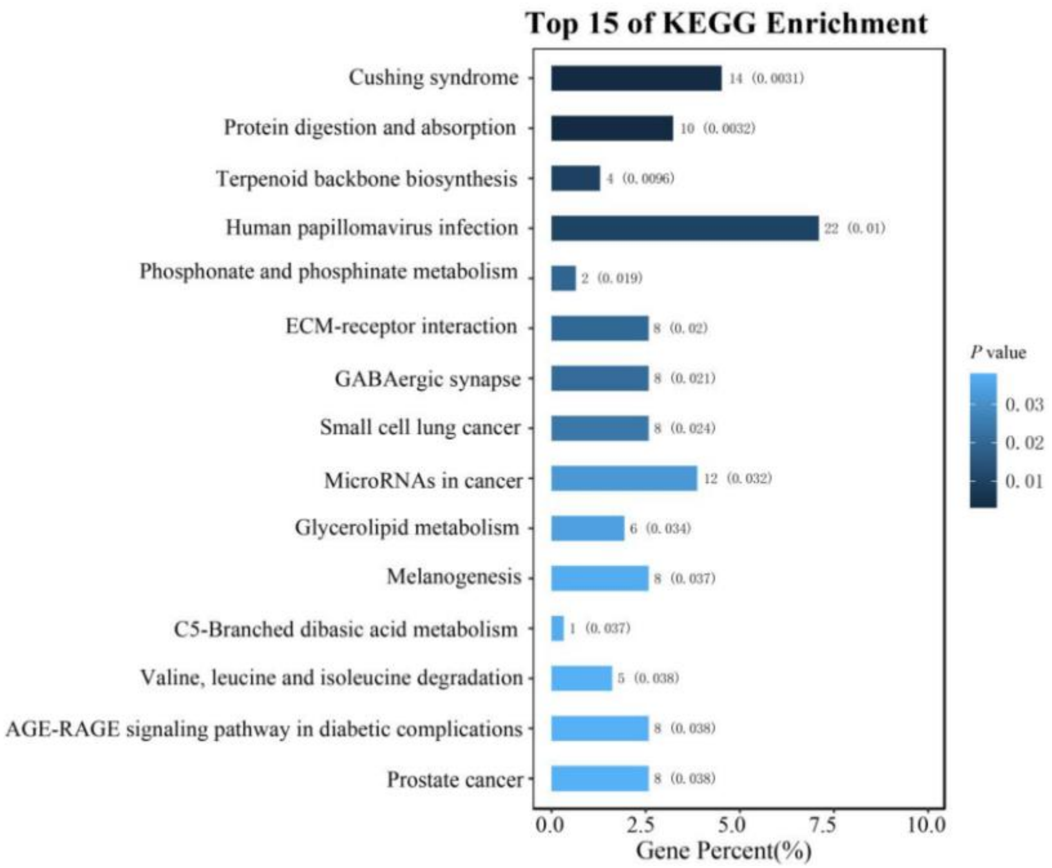

Table S6 GO enrichment analysis of Sichuan White geese

GroupID	Category	Term	Description	LogP	*P* Vaue	Log(q-value)	InTerm_InList
1_Summary	GO Biological Processes	GO:0051249	regulation of lymphocyte activation	-5.42	3.81E-06	-1.224	28/-
1_Member	GO Biological Processes	GO:0051249	regulation of lymphocyte activation	-5.42	3.81E-06	-1.224	15/506
1_Member	GO Biological Processes	GO:0002694	regulation of leukocyte activation	-4.78	1.66E-05	-1.130	15/573
1_Member	GO Biological Processes	GO:0050863	regulation of T cell activation	-4.69	2.05E-05	-1.118	12/382
1_Member	GO Biological Processes	GO:0050865	regulation of cell activation	-4.31	4.93E-05	-0.943	15/630
1_Member	GO Biological Processes	GO:0045580	regulation of T cell differentiation	-4.28	5.23E-05	-0.943	8/183
1_Member	GO Biological Processes	GO:1903706	regulation of hemopoiesis	-4.25	5.64E-05	-0.943	12/424
1_Member	GO Biological Processes	GO:1902105	regulation of leukocyte differentiation	-3.89	1.29E-04	-0.849	10/328
1_Member	GO Biological Processes	GO:0045619	regulation of lymphocyte differentiation	-3.80	1.60E-04	-0.846	8/215
1_Member	GO Biological Processes	GO:0030098	lymphocyte differentiation	-3.62	2.39E-04	-0.756	9/289

1_Member	GO Biological Processes	GO:0002521	leukocyte differentiation	-3.62	2.39E-04	-0.756	11/423
1_Member	GO Biological Processes	GO:0030099	myeloid cell differentiation	-3.61	2.45E-04	-0.756	9/290
1_Member	GO Biological Processes	GO:0002363	alpha-beta T cell lineage commitment	-3.50	3.14E-04	-0.746	3/19
1_Member	GO Biological Processes	GO:0030217	T cell differentiation	-3.49	3.26E-04	-0.746	7/181
1_Member	GO Biological Processes	GO:0045058	T cell selection	-3.46	3.47E-04	-0.746	4/47
1_Member	GO Biological Processes	GO:0030097	hemopoiesis	-3.39	4.06E-04	-0.746	14/683
1_Member	GO Biological Processes	GO:0043369	CD4-positive or CD8-positive, alpha-beta T cell lineage commitment	-3.37	4.27E-04	-0.746	3/21
1_Member	GO Biological Processes	GO:0051251	positive regulation of lymphocyte activation	-3.22	5.98E-04	-0.688	9/328
1_Member	GO Biological Processes	GO:1903131	mononuclear cell differentiation	-3.19	6.39E-04	-0.680	9/331
1_Member	GO Biological Processes	GO:0046649	lymphocyte activation	-3.18	6.65E-04	-0.669	11/478
1_Member	GO Biological Processes	GO:0001775	cell activation	-3.13	7.38E-04	-0.659	14/726
1_Member	GO Biological Processes	GO:0002573	myeloid leukocyte differentiation	-3.12	7.56E-04	-0.659	6/151
1_Member	GO Biological Processes	GO:0002360	T cell lineage commitment	-3.04	9.10E-04	-0.635	3/27
1_Member	GO Biological Processes	GO:0002696	positive regulation of leukocyte activation	-2.92	1.20E-03	-0.592	9/362
1_Member	GO Biological Processes	GO:0045321	leukocyte activation	-2.88	1.31E-03	-0.566	12/601
1_Member	GO Biological Processes	GO:0050867	positive regulation of cell activation	-2.78	1.67E-03	-0.502	9/380
1_Member	GO Biological Processes	GO:0043368	positive T cell selection	-2.75	1.79E-03	-0.481	3/34
1_Member	GO Biological Processes	GO:0046637	regulation of alpha-beta T cell differentiation	-2.72	1.93E-03	-0.474	4/74
1_Member	GO Biological Processes	GO:0042110	T cell activation	-2.69	2.06E-03	-0.462	8/318
1_Member	GO Biological Processes	GO:0046632	alpha-beta T cell differentiation	-2.67	2.13E-03	-0.456	4/76
1_Member	GO Biological Processes	GO:0002709	regulation of T cell mediated immunity	-2.26	5.50E-03	-0.239	4/99
1_Member	GO Biological Processes	GO:1903037	regulation of leukocyte cell-cell adhesion	-2.21	6.20E-03	-0.212	8/382
1_Member	GO Biological Processes	GO:0002697	regulation of immune effector process	-2.19	6.49E-03	-0.204	8/385
1_Member	GO Biological Processes	GO:0046631	alpha-beta T cell activation	-2.17	6.75E-03	-0.192	4/105
1_Member	GO Biological Processes	GO:0043367	CD4-positive, alpha-beta T cell differentiation	-2.15	7.06E-03	-0.181	3/55
1_Member	GO Biological Processes	GO:0043370	regulation of CD4-positive, alpha-beta T cell differentiation	-2.13	7.42E-03	-0.180	3/56
1_Member	KEGG Pathway	hsa04659	Th17 cell differentiation	-2.13	7.45E-03	-0.180	4/108
1_Member	GO Biological Processes	GO:0046634	regulation of alpha-beta T cell activation	-2.03	9.25E-03	-0.127	4/115
1_Member	GO Biological Processes	GO:0050870	positive regulation of T cell activation	-2.02	9.64E-03	-0.117	6/253
2_Summary	GO Biological Processes	GO:0097435	supramolecular fiber organization	-5.24	5.82E-06	-1.224	16/-
2_Member	GO Biological Processes	GO:0097435	supramolecular fiber organization	-5.24	5.82E-06	-1.224	16/592
2_Member	GO Biological Processes	GO:0045109	intermediate filament organization	-2.74	1.83E-03	-0.479	4/73
2_Member	KEGG Pathway	hsa04915	Estrogen signaling pathway	-2.52	3.01E-03	-0.352	5/137
2_Member	GO Biological Processes	GO:0045104	intermediate filament cytoskeleton organization	-2.36	4.41E-03	-0.271	4/93
2_Member	GO Biological Processes	GO:0045103	intermediate filament-based process	-2.34	4.58E-03	-0.271	4/94
2_Member	KEGG Pathway	hsa05150	Staphylococcus aureus infection	-2.31	4.93E-03	-0.262	4/96
3_Summary	GO Biological Processes	GO:0042542	response to hydrogen peroxide	-5.09	8.05E-06	-1.224	22/-
3_Member	GO Biological Processes	GO:0042542	response to hydrogen peroxide	-5.09	8.05E-06	-1.224	7/101
3_Member	GO Biological Processes	GO:0000302	response to reactive oxygen species	-4.33	4.65E-05	-0.943	8/180
3_Member	GO Biological Processes	GO:0070301	cellular response to hydrogen peroxide	-3.93	1.16E-04	-0.849	5/67
3_Member	GO Biological Processes	GO:0043525	positive regulation of neuron apoptotic process	-3.87	1.34E-04	-0.849	5/69
3_Member	GO Biological Processes	GO:0071731	response to nitric oxide	-3.43	3.68E-04	-0.746	3/20
3_Member	GO Biological Processes	GO:0034614	cellular response to reactive oxygen species	-3.43	3.72E-04	-0.746	6/132
3_Member	GO Biological Processes	GO:0062197	cellular response to chemical stress	-3.07	8.43E-04	-0.656	8/276
3_Member	GO Biological Processes	GO:0051402	neuron apoptotic process	-3.01	9.75E-04	-0.632	5/106
3_Member	GO Biological Processes	GO:0006979	response to oxidative stress	-2.89	1.29E-03	-0.567	9/366
3_Member	GO Biological Processes	GO:0070227	lymphocyte apoptotic process	-2.71	1.95E-03	-0.474	3/35
3_Member	KEGG Pathway	hsa05131	Shigellosis	-2.70	2.01E-03	-0.467	7/247

3_Member	KEGG Pathway	hsa04210	Apoptosis	-2.55	2.83E-03	-0.377	5/135
3_Member	GO Biological Processes	GO:0071887	leukocyte apoptotic process	-2.40	4.02E-03	-0.285	3/45
3_Member	KEGG Pathway	hsa05170	Human immunodeficiency virus 1 infection	-2.38	4.18E-03	-0.279	6/212
3_Member	GO Biological Processes	GO:0010035	response to inorganic substance	-2.33	4.64E-03	-0.270	10/527
3_Member	GO Biological Processes	GO:0034599	cellular response to oxidative stress	-2.28	5.22E-03	-0.252	6/222
3_Member	KEGG Pathway	hsa04064	NF-kappa B signaling pathway	-2.17	6.75E-03	-0.192	4/105
3_Member	GO Biological Processes	GO:0097190	apoptotic signaling pathway	-2.14	7.27E-03	-0.180	7/313
3_Member	GO Biological Processes	GO:0043523	regulation of neuron apoptotic process	-2.09	8.16E-03	-0.154	6/244
4_Summary	KEGG Pathway	hsa05132	Salmonella infection	-4.93	1.19E-05	-1.181	16/-
4_Member	KEGG Pathway	hsa05132	Salmonella infection	-4.93	1.19E-05	-1.181	10/247
4_Member	GO Molecular Functions	GO:0019904	protein domain specific binding	-2.59	2.59E-03	-0.403	12/653
5_Summary	GO Biological Processes	GO:0048762	mesenchymal cell differentiation	-4.44	3.66E-05	-0.943	27/-
5_Member	GO Biological Processes	GO:0048762	mesenchymal cell differentiation	-4.44	3.66E-05	-0.943	8/174
5_Member	GO Biological Processes	GO:0072009	nephron epithelium development	-3.88	1.32E-04	-0.849	6/109
5_Member	GO Biological Processes	GO:0001822	kidney development	-3.47	3.38E-04	-0.746	9/303
5_Member	GO Biological Processes	GO:0001656	metanephros development	-3.43	3.76E-04	-0.746	5/86
5_Member	GO Biological Processes	GO:0060485	mesenchyme development	-3.39	4.08E-04	-0.746	8/247
5_Member	GO Biological Processes	GO:0072001	renal system development	-3.37	4.28E-04	-0.746	9/313
5_Member	GO Biological Processes	GO:0061326	renal tubule development	-3.33	4.64E-04	-0.724	5/90
5_Member	GO Biological Processes	GO:0072006	nephron development	-3.31	4.89E-04	-0.724	6/139
5_Member	GO Biological Processes	GO:0072073	kidney epithelium development	-3.29	5.08E-04	-0.717	6/140
5_Member	GO Biological Processes	GO:0010560	positive regulation of glycoprotein biosynthetic process	-3.25	5.63E-04	-0.688	3/23
5_Member	GO Biological Processes	GO:0048599	oocyte development	-3.23	5.92E-04	-0.688	4/54
5_Member	GO Biological Processes	GO:0010559	regulation of glycoprotein biosynthetic process	-3.20	6.34E-04	-0.680	4/55
5_Member	GO Biological Processes	GO:0009994	oocyte differentiation	-3.11	7.76E-04	-0.659	4/58
5_Member	GO Biological Processes	GO:0032835	glomerulus development	-3.11	7.76E-04	-0.659	4/58
5_Member	GO Biological Processes	GO:1903020	positive regulation of glycoprotein metabolic process	-3.09	8.13E-04	-0.659	3/26
5_Member	GO Biological Processes	GO:1903018	regulation of glycoprotein metabolic process	-3.03	9.39E-04	-0.635	4/61
5_Member	GO Biological Processes	GO:0007292	female gamete generation	-3.02	9.57E-04	-0.635	6/158
5_Member	GO Biological Processes	GO:0033077	T cell differentiation in thymus	-2.95	1.12E-03	-0.614	4/64
5_Member	GO Biological Processes	GO:0031069	hair follicle morphogenesis	-2.78	1.64E-03	-0.505	3/33
5_Member	GO Biological Processes	GO:0048730	epidermis morphogenesis	-2.64	2.29E-03	-0.435	3/37
5_Member	GO Biological Processes	GO:0001942	hair follicle development	-2.59	2.56E-03	-0.404	4/80
5_Member	GO Biological Processes	GO:0022404	molting cycle process	-2.51	3.06E-03	-0.352	4/84
5_Member	GO Biological Processes	GO:0022405	hair cycle process	-2.51	3.06E-03	-0.352	4/84
5_Member	GO Biological Processes	GO:0072080	nephron tubule development	-2.50	3.19E-03	-0.344	4/85
5_Member	GO Biological Processes	GO:0090596	sensory organ morphogenesis	-2.44	3.64E-03	-0.309	7/275
5_Member	GO Biological Processes	GO:0061005	cell differentiation involved in kidney development	-2.34	4.55E-03	-0.271	3/47
5_Member	GO Biological Processes	GO:0042303	molting cycle	-2.31	4.93E-03	-0.262	4/96
5_Member	GO Biological Processes	GO:0042633	hair cycle	-2.31	4.93E-03	-0.262	4/96
5_Member	GO Biological Processes	GO:0048538	thymus development	-2.29	5.11E-03	-0.258	3/49
5_Member	KEGG Pathway	hsa05215	Prostate cancer	-2.29	5.11E-03	-0.258	4/97
5_Member	GO Biological Processes	GO:0048592	eye morphogenesis	-2.24	5.81E-03	-0.223	5/160
5_Member	GO Biological Processes	GO:0001654	eye development	-2.21	6.11E-03	-0.213	8/381
5_Member	GO Biological Processes	GO:0048477	oogenesis	-2.20	6.32E-03	-0.208	4/103
5_Member	GO Biological Processes	GO:0060070	canonical Wnt signaling pathway	-2.20	6.32E-03	-0.208	4/103
5_Member	GO Biological Processes	GO:0150063	visual system development	-2.19	6.49E-03	-0.204	8/385
5_Member	GO Biological Processes	GO:0048880	sensory system development	-2.15	7.09E-03	-0.180	8/391

5_Member	GO Biological Processes	GO:0009948	anterior/posterior axis specification	-2.07	8.56E-03	-0.145		3/59
5_Member	GO Biological Processes	GO:0007423	sensory organ development	-2.05	8.95E-03	-0.132		10/581
5_Member	GO Biological Processes	GO:0098773	skin epidermis development	-2.03	9.25E-03	-0.127		4/115
6_Summary	GO Molecular Functions	GO:0016301	kinase activity	-4.17	6.76E-05	-0.936		20/-
6_Member	GO Molecular Functions	GO:0016301	kinase activity	-4.17	6.76E-05	-0.936		16/725
6_Member	GO Molecular Functions	GO:0016773	phosphotransferase activity, alcohol group as acceptor	-4.00	9.93E-05	-0.849		15/671
6_Member	GO Molecular Functions	GO:0004674	protein serine/threonine kinase activity	-2.99	1.02E-03	-0.626		10/427
6_Member	GO Biological Processes	GO:0016310	phosphorylation	-2.69	2.05E-03	-0.462		13/721
6_Member	GO Molecular Functions	GO:0004672	protein kinase activity	-2.61	2.43E-03	-0.419		11/563
6_Member	GO Biological Processes	GO:0018193	peptidyl-amino acid modification	-2.37	4.23E-03	-0.279		10/520
6_Member	GO Molecular Functions	GO:0106310	protein serine kinase activity	-2.34	4.60E-03	-0.271		8/363
6_Member	GO Biological Processes	GO:0018209	peptidyl-serine modification	-2.08	8.40E-03	-0.146		5/175
7_Summary	KEGG Pathway	hsa04070	Phosphatidylinositol signaling system	-4.16	6.89E-05	-0.936		17/-
7_Member	KEGG Pathway	hsa04070	Phosphatidylinositol signaling system	-4.16	6.89E-05	-0.936		6/97
7_Member	GO Biological Processes	GO:0046854	phosphatidylinositol phosphate biosynthetic process	-3.97	1.08E-04	-0.849		5/66
7_Member	KEGG Pathway	hsa00562	Inositol phosphate metabolism	-3.76	1.75E-04	-0.825		5/73
7_Member	GO Molecular Functions	GO:0001727	lipid kinase activity	-3.69	2.04E-04	-0.775		4/41
7_Member	GO Biological Processes	GO:0006661	phosphatidylinositol biosynthetic process	-3.54	2.90E-04	-0.746		6/126
7_Member	GO Biological Processes	GO:0008610	lipid biosynthetic process	-3.46	3.48E-04	-0.746		13/593
7_Member	GO Biological Processes	GO:0036092	phosphatidylinositol-3-phosphate biosynthetic process	-3.31	4.92E-04	-0.724		3/22
7_Member	GO Biological Processes	GO:0090407	organophosphate biosynthetic process	-3.26	5.54E-04	-0.688		12/544
7_Member	GO Biological Processes	GO:0046474	glycerophospholipid biosynthetic process	-3.23	5.93E-04	-0.688		7/200
7_Member	GO Molecular Functions	GO:0052742	phosphatidylinositol kinase activity	-3.09	8.13E-04	-0.659		3/26
7_Member	GO Biological Processes	GO:0046488	phosphatidylinositol metabolic process	-3.02	9.57E-04	-0.635		6/158
7_Member	GO Biological Processes	GO:0045017	glycerolipid biosynthetic process	-2.96	1.09E-03	-0.619		7/222
7_Member	GO Biological Processes	GO:0008654	phospholipid biosynthetic process	-2.77	1.71E-03	-0.498		7/240
7_Member	GO Biological Processes	GO:0006650	glycerophospholipid metabolic process	-2.31	4.94E-03	-0.262		7/291
8_Summary	GO Molecular Functions	GO:0019903	protein phosphatase binding	-4.14	7.28E-05	-0.936		15/-
8_Member	GO Molecular Functions	GO:0019903	protein phosphatase binding	-4.14	7.28E-05	-0.936		7/142
8_Member	GO Molecular Functions	GO:0019902	phosphatase binding	-3.37	4.23E-04	-0.746		7/189
8_Member	KEGG Pathway	hsa04217	Necroptosis	-3.00	9.89E-04	-0.630		6/159
8_Member	GO Molecular Functions	GO:0051721	protein phosphatase 2A binding	-2.95	1.12E-03	-0.614		3/29
8_Member	KEGG Pathway	hsa04933	AGE-RAGE signaling pathway in diabetic complications	-2.24	5.69E-03	-0.230		4/100
8_Member	GO Molecular Functions	GO:0044389	ubiquitin-like protein ligase binding	-2.06	8.70E-03	-0.141		7/324
9_Summary	GO Cellular Components	GO:0043296	apical junction complex	-3.99	1.03E-04	-0.849		14/-
9_Member	GO Cellular Components	GO:0043296	apical junction complex	-3.99	1.03E-04	-0.849		7/150
9_Member	GO Cellular Components	GO:0019898	extrinsic component of membrane	-3.55	2.85E-04	-0.746		7/177
9_Member	GO Cellular Components	GO:0005923	bicellular tight junction	-3.54	2.90E-04	-0.746		6/126
9_Member	GO Cellular Components	GO:0070160	tight junction	-3.45	3.57E-04	-0.746		6/131
9_Member	GO Cellular Components	GO:0016342	catenin complex	-2.86	1.37E-03	-0.561		3/31
9_Member	GO Biological Processes	GO:0045216	cell-cell junction organization	-2.77	1.71E-03	-0.498		6/177
9_Member	GO Biological Processes	GO:0007043	cell-cell junction assembly	-2.68	2.10E-03	-0.458		5/126
9_Member	GO Cellular Components	GO:0005912	adherens junction	-2.64	2.32E-03	-0.434		6/188
9_Member	GO Biological Processes	GO:0070830	bicellular tight junction assembly	-2.27	5.41E-03	-0.244		3/50
9_Member	GO Biological Processes	GO:0034332	adherens junction organization	-2.22	6.04E-03	-0.214		3/52
9_Member	GO Cellular Components	GO:0019897	extrinsic component of plasma membrane	-2.21	6.10E-03	-0.213		4/102
9_Member	GO Biological Processes	GO:0120192	tight junction assembly	-2.13	7.42E-03	-0.180		3/56

9_Member	GO Biological Processes	GO:0043297	apical junction assembly	-2.09	8.17E-03	-0.154	3/58
9_Member	GO Biological Processes	GO:0120193	tight junction organization	-2.07	8.56E-03	-0.145	3/59
10_Summary	GO Biological Processes	GO:0060322	head development	-3.82	1.52E-04	-0.846	32/-
10_Member	GO Biological Processes	GO:0060322	head development	-3.82	1.52E-04	-0.846	16/778
10_Member	GO Biological Processes	GO:0009952	anterior/posterior pattern specification	-3.81	1.55E-04	-0.846	8/214
10_Member	GO Biological Processes	GO:0007420	brain development	-3.61	2.43E-04	-0.756	15/729
10_Member	GO Biological Processes	GO:0007389	pattern specification process	-3.32	4.76E-04	-0.724	11/459
10_Member	GO Biological Processes	GO:0035282	segmentation	-3.16	6.84E-04	-0.663	5/98
10_Member	GO Biological Processes	GO:0003002	regionalization	-3.11	7.76E-04	-0.659	10/412
10_Member	GO Biological Processes	GO:0043009	chordate embryonic development	-3.09	8.17E-04	-0.659	13/650
10_Member	GO Biological Processes	GO:0001756	somitogenesis	-3.03	9.39E-04	-0.635	4/61
10_Member	GO Biological Processes	GO:0009792	embryo development ending in birth or egg hatching	-2.96	1.09E-03	-0.619	13/671
10_Member	GO Biological Processes	GO:0048568	embryonic organ development	-2.81	1.55E-03	-0.518	10/452
10_Member	GO Biological Processes	GO:0001701	in utero embryonic development	-2.66	2.17E-03	-0.453	9/395
10_Member	GO Biological Processes	GO:0061053	somite development	-2.61	2.45E-03	-0.419	4/79
10_Member	GO Biological Processes	GO:0007507	heart development	-2.10	7.89E-03	-0.164	10/570
11_Summary	GO Biological Processes	GO:0006970	response to osmotic stress	-3.81	1.53E-04	-0.846	12/-
11_Member	GO Biological Processes	GO:0006970	response to osmotic stress	-3.81	1.53E-04	-0.846	5/71
11_Member	GO Biological Processes	GO:0070293	renal absorption	-2.78	1.64E-03	-0.505	3/33
11_Member	GO Biological Processes	GO:0071260	cellular response to mechanical stimulus	-2.74	1.83E-03	-0.479	4/73
11_Member	GO Biological Processes	GO:0071470	cellular response to osmotic stress	-2.42	3.77E-03	-0.304	3/44
11_Member	GO Biological Processes	GO:0009612	response to mechanical stimulus	-2.34	4.58E-03	-0.271	6/216
11_Member	GO Biological Processes	GO:0071214	cellular response to abiotic stimulus	-2.10	7.89E-03	-0.164	7/318
11_Member	GO Biological Processes	GO:0104004	cellular response to environmental stimulus	-2.10	7.89E-03	-0.164	7/318
12_Summary	GO Cellular Components	GO:0005635	nuclear envelope	-3.60	2.52E-04	-0.756	12/-
12_Member	GO Cellular Components	GO:0005635	nuclear envelope	-3.60	2.52E-04	-0.756	12/498
12_Member	GO Cellular Components	GO:0031965	nuclear membrane	-3.37	4.28E-04	-0.746	9/313
13_Summary	GO Biological Processes	GO:0061458	reproductive system development	-3.49	3.22E-04	-0.746	25/-
13_Member	GO Biological Processes	GO:0061458	reproductive system development	-3.49	3.22E-04	-0.746	9/301
13_Member	GO Biological Processes	GO:0007548	sex differentiation	-3.02	9.46E-04	-0.635	8/281
13_Member	GO Biological Processes	GO:0010506	regulation of autophagy	-2.96	1.09E-03	-0.619	9/357
13_Member	GO Biological Processes	GO:0048608	reproductive structure development	-2.87	1.35E-03	-0.561	8/297
13_Member	GO Biological Processes	GO:0007346	regulation of mitotic cell cycle	-2.87	1.35E-03	-0.561	11/522
13_Member	GO Biological Processes	GO:0048873	homeostasis of number of cells within a tissue	-2.71	1.95E-03	-0.474	3/35
13_Member	GO Biological Processes	GO:2000045	regulation of G1/S transition of mitotic cell cycle	-2.67	2.14E-03	-0.456	6/185
13_Member	GO Biological Processes	GO:2000134	negative regulation of G1/S transition of mitotic cell cycle	-2.50	3.19E-03	-0.344	4/85
13_Member	GO Biological Processes	GO:1902806	regulation of cell cycle G1/S phase transition	-2.39	4.09E-03	-0.284	6/211
13_Member	GO Biological Processes	GO:1902807	negative regulation of cell cycle G1/S phase transition	-2.34	4.58E-03	-0.271	4/94
13_Member	GO Biological Processes	GO:0001894	tissue homeostasis	-2.31	4.89E-03	-0.262	6/219
13_Member	GO Biological Processes	GO:0060249	anatomical structure homeostasis	-2.31	4.89E-03	-0.262	6/219
13_Member	GO Biological Processes	GO:0046545	development of primary female sexual characteristics	-2.29	5.11E-03	-0.258	4/97
13_Member	GO Biological Processes	GO:0045137	development of primary sexual characteristics	-2.21	6.18E-03	-0.212	6/230
13_Member	GO Biological Processes	GO:0001541	ovarian follicle development	-2.20	6.37E-03	-0.207	3/53
13_Member	GO Biological Processes	GO:0045930	negative regulation of mitotic cell cycle	-2.19	6.44E-03	-0.204	6/232
13_Member	GO Biological Processes	GO:0046660	female sex differentiation	-2.07	8.45E-03	-0.146	4/112
14_Summary	GO Cellular Components	GO:0005938	cell cortex	-3.41	3.90E-04	-0.746	9/-
14_Member	GO Cellular Components	GO:0005938	cell cortex	-3.41	3.90E-04	-0.746	9/309

15_Summary	GO Biological Processes	GO:0032986	protein-DNA complex disassembly	-3.37	4.27E-04	-0.746	3/-
15_Member	GO Biological Processes	GO:0032986	protein-DNA complex disassembly	-3.37	4.27E-04	-0.746	3/21
16_Summary	GO Biological Processes	GO:0046328	regulation of JNK cascade	-3.33	4.71E-04	-0.724	24/-
16_Member	GO Biological Processes	GO:0046328	regulation of JNK cascade	-3.33	4.71E-04	-0.724	6/138
16_Member	GO Biological Processes	GO:0046330	positive regulation of JNK cascade	-3.23	5.94E-04	-0.688	5/95
16_Member	GO Biological Processes	GO:0001819	positive regulation of cytokine production	-3.02	9.58E-04	-0.635	11/500
16_Member	GO Biological Processes	GO:0043122	regulation of canonical NF-kappaB signal transduction	-2.35	4.50E-03	-0.271	7/286
16_Member	GO Molecular Functions	GO:0032813	tumor necrosis factor receptor superfamily binding	-2.29	5.11E-03	-0.258	3/49
16_Member	GO Biological Processes	GO:0043410	positive regulation of MAPK cascade	-2.15	7.15E-03	-0.180	9/475
16_Member	KEGG Pathway	hsa04620	Toll-like receptor signaling pathway	-2.13	7.45E-03	-0.180	4/108
16_Member	GO Molecular Functions	GO:0035591	signaling adaptor activity	-2.07	8.45E-03	-0.146	4/112
16_Member	GO Biological Processes	GO:0043408	regulation of MAPK cascade	-2.06	8.65E-03	-0.142	11/669
17_Summary	GO Molecular Functions	GO:0042803	protein homodimerization activity	-3.20	6.28E-04	-0.680	18/-
17_Member	GO Molecular Functions	GO:0042803	protein homodimerization activity	-3.20	6.28E-04	-0.680	14/714
17_Member	GO Biological Processes	GO:0051607	defense response to virus	-2.47	3.43E-03	-0.324	7/272
17_Member	GO Biological Processes	GO:0009615	response to virus	-2.23	5.92E-03	-0.218	8/379
18_Summary	GO Biological Processes	GO:0050678	regulation of epithelial cell proliferation	-3.15	7.07E-04	-0.659	20/-
18_Member	GO Biological Processes	GO:0050678	regulation of epithelial cell proliferation	-3.15	7.07E-04	-0.659	10/407
18_Member	GO Biological Processes	GO:0010464	regulation of mesenchymal cell proliferation	-2.78	1.64E-03	-0.505	3/33
18_Member	GO Biological Processes	GO:1901699	cellular response to nitrogen compound	-2.71	1.96E-03	-0.474	12/631
18_Member	GO Biological Processes	GO:0071417	cellular response to organonitrogen compound	-2.66	2.21E-03	-0.448	11/556
18_Member	GO Biological Processes	GO:0045601	regulation of endothelial cell differentiation	-2.37	4.28E-03	-0.279	3/46
18_Member	GO Biological Processes	GO:0030857	negative regulation of epithelial cell differentiation	-2.15	7.06E-03	-0.181	3/55
18_Member	GO Biological Processes	GO:0030856	regulation of epithelial cell differentiation	-2.12	7.64E-03	-0.171	5/171
19_Summary	GO Molecular Functions	GO:0051015	actin filament binding	-3.13	7.47E-04	-0.659	11/-
19_Member	GO Molecular Functions	GO:0051015	actin filament binding	-3.13	7.47E-04	-0.659	7/208
19_Member	GO Cellular Components	GO:0001725	stress fiber	-2.50	3.19E-03	-0.344	4/85
19_Member	GO Cellular Components	GO:0097517	contractile actin filament bundle	-2.50	3.19E-03	-0.344	4/85
19_Member	GO Cellular Components	GO:0015629	actin cytoskeleton	-2.43	3.69E-03	-0.306	10/510
19_Member	GO Molecular Functions	GO:0003779	actin binding	-2.39	4.03E-03	-0.285	9/434
19_Member	GO Cellular Components	GO:0042641	actomyosin	-2.39	4.08E-03	-0.284	4/91
19_Member	GO Cellular Components	GO:0032432	actin filament bundle	-2.32	4.75E-03	-0.264	4/95
20_Summary	GO Biological Processes	GO:0000165	MAPK cascade	-3.11	7.68E-04	-0.659	7/-
20_Member	GO Biological Processes	GO:0000165	MAPK cascade	-3.11	7.68E-04	-0.659	7/209
20_Member	GO Molecular Functions	GO:0004709	MAP kinase kinase kinase activity	-3.09	8.13E-04	-0.659	3/26

Table S7 GO enrichment analysis of the Sichuan White Geese (continuation of the table)

Term	Genes
GO:0051249	472,596,1236,1326,1499,2648,3662,4615,6605,6935,30009,54440,56940,79576,196528,6772,8737,85477,7155,6776,5305,10006,81027,6845,54518,8527,9390,79132
GO:0051249	472,596,1236,1326,1499,2648,3662,4615,6605,6935,30009,54440,56940,79576,196528
GO:0002694	472,596,1236,1326,1499,2648,3662,4615,6605,6935,30009,54440,56940,79576,196528
GO:0050863	1236,1326,1499,2648,3662,6605,6935,30009,54440,56940,79576,196528
GO:0050865	472,596,1236,1326,1499,2648,3662,4615,6605,6935,30009,54440,56940,79576,196528
GO:0045580	2648,3662,6605,6935,30009,54440,79576,196528
GO:1903706	1499,2648,3662,6605,6772,6935,8737,30009,54440,79576,85477,196528
GO:1902105	1499,2648,3662,6605,6935,8737,30009,54440,79576,196528

GO:0045619	2648,3662,6605,6935,30009,54440,79576,196528
GO:0030098	472,596,1236,1499,3662,7155,30009,54440,79576
GO:0002521	472,596,1236,1499,3662,4615,6776,7155,30009,54440,79576
GO:0030099	1236,1499,3662,4615,5305,6776,10006,79576,81027
GO:0002363	596,3662,30009
GO:0030217	596,1236,1499,3662,30009,54440,79576
GO:0045058	596,1236,3662,30009
GO:0030097	472,596,1236,1499,3662,4615,5305,6776,7155,10006,30009,54440,79576,81027
GO:0043369	596,3662,30009
GO:0051251	596,1236,1326,4615,6605,30009,54440,79576,196528
GO:1903131	472,596,1236,1499,3662,7155,30009,54440,79576
GO:0046649	472,596,1236,1499,3662,6845,7155,30009,54440,54518,79576
GO:0001775	472,596,1236,1499,3662,4615,6845,7155,8527,30009,54440,54518,79576,81027
GO:0002573	1236,1499,3662,4615,6776,79576
GO:0002360	596,3662,30009
GO:0002696	596,1236,1326,4615,6605,30009,54440,79576,196528
GO:0045321	472,596,1236,1499,3662,4615,6845,7155,30009,54440,54518,79576
GO:0050867	596,1236,1326,4615,6605,30009,54440,79576,196528
GO:0043368	596,3662,30009
GO:0046637	3662,30009,54440,79576
GO:0042110	596,1236,1499,3662,30009,54440,54518,79576
GO:0046632	596,3662,30009,54440
GO:0002709	9390,30009,54440,56940
GO:1903037	1236,1326,6605,30009,54440,56940,79576,196528
GO:0002697	3662,4615,6845,9390,30009,54440,56940,79132
GO:0046631	596,3662,30009,54440
GO:0043367	3662,30009,54440
GO:0043370	3662,30009,54440
hsa04659	3662,6772,6776,30009
GO:0046634	3662,30009,54440,79576
GO:0050870	1236,1326,6605,54440,79576,196528
GO:0097435	596,2296,3837,3859,3866,3872,8636,8737,10006,10529,10609,25984,57477,81027,85477,129446
GO:0097435	596,2296,3837,3859,3866,3872,8636,8737,10006,10529,10609,25984,57477,81027,85477,129446
GO:0045109	3859,3866,3872,25984
hsa04915	596,3859,3866,3872,25984
GO:0045104	3859,3866,3872,25984
GO:0045103	3859,3866,3872,25984
hsa05150	3859,3866,3872,25984
GO:0042542	358,596,6772,7155,7703,8737,9131,472,840,1499,1236,5269,9943,5459,4615,10325,23157,84812,8065,85417,1910,54407
GO:0042542	358,596,6772,7155,7703,8737,9131
GO:0000302	358,472,596,6772,7155,7703,8737,9131
GO:0070301	358,7155,7703,8737,9131
GO:0043525	472,840,1499,8737,9131
GO:0071731	358,1236,9131
GO:0034614	358,472,7155,7703,8737,9131
GO:0062197	358,472,5269,7155,7703,8737,9131,9943
GO:0051402	472,596,840,5459,9131
GO:0006979	358,472,596,6772,7155,7703,8737,9131,9943
GO:0070227	596,840,8737

hsa05131	472,596,4615,8737,10325,23157,84812
hsa04210	472,596,840,8737,9131
GO:0071887	596,840,8737
hsa05170	472,596,4615,8065,8737,85417
GO:0010035	358,596,1236,1910,6772,7155,7703,8737,9131,54407
GO:0034599	358,472,7155,7703,8737,9131
hsa04064	472,596,4615,8737
GO:0097190	472,596,1499,7703,8065,8737,9131
GO:0043523	472,596,840,1499,8737,9131
hsa05132	596,840,1499,4615,5286,5878,6934,8737,10006,81027,153,3757,3837,30837,84632,143098
hsa05132	596,840,1499,4615,5286,5878,6934,8737,10006,81027
GO:0019904	153,596,1499,3757,3837,4615,6934,8737,10006,30837,84632,143098
GO:0048762	596,1499,1910,2295,2296,6772,80320,139285,38,358,2674,1236,6934,472,56165,9210,84930,79576,3872,50814,5459,6935,140469,2916,3859,9525,1046
GO:0048762	596,1499,1910,2295,2296,6772,80320,139285
GO:0072009	38,358,596,1499,1910,2296
GO:0001822	38,358,596,1499,1910,2296,2674,6772,139285
GO:0001656	38,358,596,1499,6772
GO:0060485	596,1499,1910,2295,2296,6772,80320,139285
GO:0072001	38,358,596,1499,1910,2296,2674,6772,139285
GO:0061326	38,358,596,1499,6772
GO:0072006	38,358,596,1499,1910,2296
GO:0072073	38,358,596,1499,1910,2296
GO:0010560	1236,1499,6934
GO:0048599	472,596,1499,56165
GO:0010559	596,1236,1499,6934
GO:0009994	472,596,1499,56165
GO:0032835	358,596,1910,2296
GO:1903020	1236,1499,6934
GO:1903018	596,1236,1499,6934
GO:0007292	472,596,1499,9210,56165,84930
GO:0033077	596,1236,1499,79576
GO:0031069	596,1499,3872
GO:0048730	596,1499,3872
GO:0001942	596,1499,3872,50814
GO:0022404	596,1499,3872,50814
GO:0022405	596,1499,3872,50814
GO:0072080	38,358,596,1499
GO:0090596	358,596,1499,2295,5459,6935,140469
GO:0061005	1910,6772,139285
GO:0042303	596,1499,3872,50814
GO:0042633	596,1499,3872,50814
GO:0048538	472,596,1499
hsa05215	596,1499,6934,6935
GO:0048592	358,596,1499,2295,6935
GO:0001654	358,596,1499,2295,2296,2916,3859,6935
GO:0048477	472,596,1499,56165
GO:0060070	1499,1910,6934,9525
GO:0150063	358,596,1499,2295,2296,2916,3859,6935
GO:0048880	358,596,1499,2295,2296,2916,3859,6935

GO:0009948	1046,1499,56165
GO:0007423	358,596,1499,2295,2296,2916,3859,5459,6935,140469
GO:0098773	596,1499,3872,50814
GO:0016301	472,1326,2324,5256,5286,5305,7084,8527,8737,9943,10746,84930,91419,140469,283209,340156,2648,10609,51114,79070
GO:0016301	472,1326,2324,5256,5286,5305,7084,8527,8737,9943,10746,84930,91419,140469,283209,340156
GO:0016773	472,1326,2324,5256,5286,5305,8527,8737,9943,10746,84930,91419,140469,283209,340156
GO:0004674	472,1326,5256,8737,9943,10746,84930,91419,140469,340156
GO:0016310	472,1326,2324,5286,5305,7084,8527,8737,9943,10746,84930,140469,340156
GO:0004672	472,1326,2324,5256,8737,9943,10746,84930,91419,140469,340156
GO:0018193	472,2324,2648,8737,9943,10609,51114,79070,84930,140469
GO:0106310	472,1326,8737,9943,10746,84930,140469,340156
GO:0018209	472,8737,79070,84930,140469
hsa04070	4952,5286,5305,8527,9108,84812,472,30837,38,47,2531,23590,50814,114881,2617,7084,9390
hsa04070	4952,5286,5305,8527,9108,84812
GO:0046854	472,4952,5286,5305,30837
hsa00562	4952,5286,5305,9108,84812
GO:0001727	472,5286,5305,8527
GO:0006661	472,4952,5286,5305,9108,30837
GO:0008610	38,47,472,2531,4952,5286,5305,8527,9108,23590,30837,50814,114881
GO:0036092	472,4952,5286
GO:0090407	38,47,472,2617,4952,5286,5305,7084,8527,9108,9390,30837
GO:0046474	472,4952,5286,5305,8527,9108,30837
GO:0052742	472,5286,5305
GO:0046488	472,4952,5286,5305,9108,30837
GO:0045017	472,4952,5286,5305,8527,9108,30837
GO:0008654	472,4952,5286,5305,8527,9108,30837
GO:0006650	472,4952,5286,5305,8527,9108,30837
GO:0019903	596,1003,1499,2324,2648,6772,84930,6776,8737,9131,9525,84812,3757,8065,9349
GO:0019903	596,1003,1499,2324,2648,6772,84930
GO:0019902	596,1003,1499,2324,2648,6772,84930
hsa04217	596,6772,6776,8737,9131,9525
GO:0051721	596,6772,84930
hsa04933	596,6772,6776,84812
GO:0044389	596,1499,3757,6772,8065,8737,9349
GO:0043296	117,1003,1499,57125,57477,91862,143098,1008,2674,4615,5286,30837,129446,57608
GO:0043296	117,1003,1499,57125,57477,91862,143098
GO:0019898	1003,1008,1499,2674,4615,5286,30837
GO:0005923	117,1003,1499,57125,91862,143098
GO:0070160	117,1003,1499,57125,91862,143098
GO:0016342	1003,1008,1499
GO:0045216	1003,1008,1499,91862,129446,143098
GO:0007043	1003,1008,1499,91862,143098
GO:0005912	1003,1008,1499,57477,57608,143098
GO:0070830	1003,91862,143098
GO:0034332	1003,1008,1499
GO:0019897	1003,1008,1499,4615
GO:0120192	1003,91862,143098
GO:0043297	1003,91862,143098
GO:0120193	1003,91862,143098

GO:0060322	358,472,596,1267,1499,1662,2296,2648,6935,7155,54407,55130,57477,57512,65109,83538,1046,7703,10006,56165,4863,4952,50814,56243,123920,2295,5459,140469,196528,840,10529,129446
GO:0060322	358,472,596,1267,1499,1662,2296,2648,6935,7155,54407,55130,57477,57512,65109,83538
GO:0009952	472,1046,1499,2296,2648,7703,10006,56165
GO:0007420	358,472,596,1267,1499,2296,2648,6935,7155,54407,55130,57477,57512,65109,83538
GO:0007389	472,1046,1499,2296,2648,6935,7703,10006,55130,56165,83538
GO:0035282	472,2296,2648,10006,56165
GO:0003002	472,1046,1499,2296,2648,7703,10006,55130,56165,83538
GO:0043009	472,1046,1499,2296,2648,4863,4952,6935,7703,10006,50814,56243,123920
GO:0001756	472,2296,2648,10006
GO:0009792	472,1046,1499,2296,2648,4863,4952,6935,7703,10006,50814,56243,123920
GO:0048568	1046,1499,2295,2296,5459,6935,7703,50814,140469,196528
GO:0001701	1046,1499,2296,2648,4863,4952,7703,50814,123920
GO:0061053	472,2296,2648,10006
GO:0007507	472,840,1499,2296,2648,10529,55130,83538,129446,196528
GO:0006970	358,1831,5269,9943,91862,1910,4615,10746,54407,1499,6772,472
GO:0006970	358,1831,5269,9943,91862
GO:0070293	358,1910,9943
GO:0071260	358,4615,10746,54407
GO:0071470	358,5269,9943
GO:0009612	358,1499,4615,6772,10746,54407
GO:0071214	358,472,4615,5269,9943,10746,54407
GO:0104004	358,472,4615,5269,9943,10746,54407
GO:0005635	358,596,1003,1069,1910,3837,9525,9805,51634,84514,114881,123920
GO:0005635	358,596,1003,1069,1910,3837,9525,9805,51634,84514,114881,123920
GO:0031965	358,596,1003,1910,3837,9805,51634,114881,123920
GO:0061458	472,596,1499,2295,2296,2674,5268,54477,317719,117,5286,5305,10325,11043,51322,55763,114881,6605,9525,10217,84632,84930,196528,54440,1003
GO:0061458	472,596,1499,2295,2296,2674,5268,54477,317719
GO:0007548	117,472,596,1499,2295,2296,2674,317719
GO:0010506	472,596,5286,5305,10325,11043,51322,55763,114881
GO:0048608	472,596,1499,2295,2296,2674,5268,317719
GO:0007346	472,596,1499,2296,6605,9525,10217,51322,84632,84930,196528
GO:0048873	596,54440,317719
GO:2000045	472,596,6605,10217,51322,196528
GO:2000134	472,596,10217,51322
GO:1902806	472,596,6605,10217,51322,196528
GO:1902807	472,596,10217,51322
GO:0001894	596,1003,1499,2296,54440,317719
GO:0060249	596,1003,1499,2296,54440,317719
GO:0046545	117,472,596,2296
GO:0045137	117,472,596,2296,2674,317719
GO:0001541	472,596,2296
GO:0045930	472,596,1499,2296,10217,51322
GO:0046660	117,472,596,2296
GO:0005938	1499,2571,3927,23157,55763,57477,85477,93661,143098
GO:0005938	1499,2571,3927,23157,55763,57477,85477,93661,143098
GO:0032986	6605,9349,196528
GO:0032986	6605,9349,196528
GO:0046328	1236,2324,4615,8737,56940,91862,2648,3662,6772,30009,54440,79132,84632,1910,11043,28511,1499,3487,57608,768211,1326,10006,143098,10251
GO:0046328	1236,2324,4615,8737,56940,91862

GO:0046330	1236,2324,4615,8737,56940
GO:0001819	1236,2324,2648,3662,4615,6772,8737,30009,54440,79132,84632
GO:0043122	1236,1910,4615,6772,8737,11043,28511
GO:0032813	4615,6772,8737
GO:0043410	1236,1499,2324,3487,4615,8737,56940,57608,768211
hsa04620	1326,4615,6772,8737
GO:0035591	4615,8737,10006,143098
GO:0043408	1236,1499,2324,3487,4615,8737,10251,56940,57608,91862,768211
GO:0042803	596,2324,2916,3757,4835,5305,6772,8527,8737,9016,9525,11043,55149,730249,4615,55763,79132,30009
GO:0042803	596,2324,2916,3757,4835,5305,6772,8527,8737,9016,9525,11043,55149,730249
GO:0051607	596,4615,6772,11043,55763,79132,730249
GO:0009615	596,4615,6772,11043,30009,55763,79132,730249
GO:0050678	1499,1910,2324,5268,6772,6776,6934,6935,57608,91862,358,840,1395,5286,7155,9131,9349,10325,1003,2296
GO:0050678	1499,1910,2324,5268,6772,6776,6934,6935,57608,91862
GO:0010464	1499,6772,6935
GO:1901699	358,840,1395,1499,5286,6772,6776,6935,7155,9131,9349,10325
GO:0071417	358,840,1395,1499,5286,6772,6776,6935,7155,9349,10325
GO:0045601	1003,1499,6935
GO:0030857	1499,6772,6935
GO:0030856	1003,1499,2296,6772,6935
GO:0051015	3927,3983,10529,57477,85477,93661,129446,56940,57584,140469,84951
GO:0051015	3927,3983,10529,57477,85477,93661,129446
GO:0001725	3983,10529,57477,129446
GO:0097517	3983,10529,57477,129446
GO:0015629	3927,3983,10529,56940,57477,57584,85477,93661,129446,140469
GO:0003779	3927,3983,10529,57477,84951,85477,93661,129446,140469
GO:0042641	3983,10529,57477,129446
GO:0032432	3983,10529,57477,129446
GO:0000165	1326,1499,3487,4615,8737,10746,51114
GO:0000165	1326,1499,3487,4615,8737,10746,51114
GO:0004709	1326,8737,10746

Table S8 GO enrichment analysis of the Sichuan White Geese (continuation of the table)

Term	Symbols
GO:0051249	ATM,BCL2,CCR7,MAP3K8,CTNNB1,KAT2A,IRF4,MYD88,SMARCE1,ZEB1,TBX21,SASH3,DUSP22,NKAP,ARID2,STAT1,RIPK1,SCIN,TOP2B,STAT5A,PIP4K2A,ABI1,TUBB1,VAMP7,APBB1IP,DGKD,SLC22A13,DHX58
GO:0051249	ATM,BCL2,CCR7,MAP3K8,CTNNB1,KAT2A,IRF4,MYD88,SMARCE1,ZEB1,TBX21,SASH3,DUSP22,NKAP,ARID2
GO:0002694	ATM,BCL2,CCR7,MAP3K8,CTNNB1,KAT2A,IRF4,MYD88,SMARCE1,ZEB1,TBX21,SASH3,DUSP22,NKAP,ARID2
GO:0050863	CCR7,MAP3K8,CTNNB1,KAT2A,IRF4,SMARCE1,ZEB1,TBX21,SASH3,DUSP22,NKAP,ARID2
GO:0050865	ATM,BCL2,CCR7,MAP3K8,CTNNB1,KAT2A,IRF4,MYD88,SMARCE1,ZEB1,TBX21,SASH3,DUSP22,NKAP,ARID2
GO:0045580	KAT2A,IRF4,SMARCE1,ZEB1,TBX21,SASH3,NKAP,ARID2
GO:1903706	CTNNB1,KAT2A,IRF4,SMARCE1,STAT1,ZEB1,RIPK1,TBX21,SASH3,NKAP,SCIN,ARID2
GO:1902105	CTNNB1,KAT2A,IRF4,SMARCE1,ZEB1,RIPK1,TBX21,SASH3,NKAP,ARID2
GO:0045619	KAT2A,IRF4,SMARCE1,ZEB1,TBX21,SASH3,NKAP,ARID2
GO:0030098	ATM,BCL2,CCR7,CTNNB1,IRF4,TOP2B,TBX21,SASH3,NKAP
GO:0002521	ATM,BCL2,CCR7,CTNNB1,IRF4,MYD88,STAT5A,TOP2B,TBX21,SASH3,NKAP
GO:0030099	CCR7,CTNNB1,IRF4,MYD88,PIP4K2A,STAT5A,ABI1,NKAP,TUBB1
GO:0002363	BCL2,IRF4,TBX21
GO:0030217	BCL2,CCR7,CTNNB1,IRF4,TBX21,SASH3,NKAP
GO:0045058	BCL2,CCR7,IRF4,TBX21
GO:0030097	ATM,BCL2,CCR7,CTNNB1,IRF4,MYD88,PIP4K2A,STAT5A,TOP2B,ABI1,TBX21,SASH3,NKAP,TUBB1
GO:0043369	BCL2,IRF4,TBX21
GO:0051251	BCL2,CCR7,MAP3K8,MYD88,SMARCE1,TBX21,SASH3,NKAP,ARID2
GO:1903131	ATM,BCL2,CCR7,CTNNB1,IRF4,TOP2B,TBX21,SASH3,NKAP
GO:0046649	ATM,BCL2,CCR7,CTNNB1,IRF4,VAMP7,TOP2B,TBX21,SASH3,APBB1IP,NKAP
GO:0001775	ATM,BCL2,CCR7,CTNNB1,IRF4,MYD88,VAMP7,TOP2B,DGKD,TBX21,SASH3,APBB1IP,NKAP,TUBB1
GO:0002573	CCR7,CTNNB1,IRF4,MYD88,STAT5A,NKAP
GO:0002360	BCL2,IRF4,TBX21
GO:0002696	BCL2,CCR7,MAP3K8,MYD88,SMARCE1,TBX21,SASH3,NKAP,ARID2
GO:0045321	ATM,BCL2,CCR7,CTNNB1,IRF4,MYD88,VAMP7,TOP2B,TBX21,SASH3,APBB1IP,NKAP
GO:0050867	BCL2,CCR7,MAP3K8,MYD88,SMARCE1,TBX21,SASH3,NKAP,ARID2
GO:0043368	BCL2,IRF4,TBX21
GO:0046637	IRF4,TBX21,SASH3,NKAP
GO:0042110	BCL2,CCR7,CTNNB1,IRF4,TBX21,SASH3,APBB1IP,NKAP
GO:0046632	BCL2,IRF4,TBX21,SASH3

GO:0002709	SLC22A13,TBX21,SASH3,DUSP22
GO:1903037	CCR7,MAP3K8,SMARCE1,TBX21,SASH3,DUSP22,NKAP,ARID2
GO:0002697	IRF4,MYD88,VAMP7,SLC22A13,TBX21,SASH3,DUSP22,DHX58
GO:0046631	BCL2,IRF4,TBX21,SASH3
GO:0043367	IRF4,TBX21,SASH3
GO:0043370	IRF4,TBX21,SASH3
hsa04659	IRF4,STAT1,STAT5A,TBX21
GO:0046634	IRF4,TBX21,SASH3,NKAP
GO:0050870	CCR7,MAP3K8,SMARCE1,SASH3,NKAP,ARID2
GO:0097435	BCL2,FOXC1,KPNB1,KRT12,KRT15,KRT17,SSNA1,RIPK1,ABI1,NEBL,P3H4,KRT23,SHROOM4,TUBB1,SCIN,XIRP2
GO:0097435	BCL2,FOXC1,KPNB1,KRT12,KRT15,KRT17,SSNA1,RIPK1,ABI1,NEBL,P3H4,KRT23,SHROOM4,TUBB1,SCIN,XIRP2
GO:0045109	KRT12,KRT15,KRT17,KRT23
hsa04915	BCL2,KRT12,KRT15,KRT17,KRT23
GO:0045104	KRT12,KRT15,KRT17,KRT23
GO:0045103	KRT12,KRT15,KRT17,KRT23
hsa05150	KRT12,KRT15,KRT17,KRT23
GO:0042542	AQP1,BCL2,STAT1,TOP2B,PCGF2,RIPK1,AIFM1,ATM,CASP7,CTNNB1,CCR7,SERPINB6,OXSR1,POU4F3,MYD88,RRAGB,SEPTIN6,PLCD4,CUL5,CCNB3,EDNRB,SLC38A2
GO:0042542	AQP1,BCL2,STAT1,TOP2B,PCGF2,RIPK1,AIFM1
GO:0000302	AQP1,ATM,BCL2,STAT1,TOP2B,PCGF2,RIPK1,AIFM1
GO:0070301	AQP1,TOP2B,PCGF2,RIPK1,AIFM1
GO:0043525	ATM,CASP7,CTNNB1,RIPK1,AIFM1
GO:0071731	AQP1,CCR7,AIFM1
GO:0034614	AQP1,ATM,TOP2B,PCGF2,RIPK1,AIFM1
GO:0062197	AQP1,ATM,SERPINB6,TOP2B,PCGF2,RIPK1,AIFM1,OXSR1
GO:0051402	ATM,BCL2,CASP7,POU4F3,AIFM1
GO:0006979	AQP1,ATM,BCL2,STAT1,TOP2B,PCGF2,RIPK1,AIFM1,OXSR1
GO:0070227	BCL2,CASP7,RIPK1
hsa05131	ATM,BCL2,MYD88,RIPK1,RRAGB,SEPTIN6,PLCD4
hsa04210	ATM,BCL2,CASP7,RIPK1,AIFM1
GO:0071887	BCL2,CASP7,RIPK1
hsa05170	ATM,BCL2,MYD88,CUL5,RIPK1,CCNB3
GO:0010035	AQP1,BCL2,CCR7,EDNRB,STAT1,TOP2B,PCGF2,RIPK1,AIFM1,SLC38A2

GO:0034599	AQP1,ATM,TOP2B,PCGF2,RIPK1,AIFM1
hsa04064	ATM,BCL2,MYD88,RIPK1
GO:0097190	ATM,BCL2,CTNNB1,PCGF2,CUL5,RIPK1,AIFM1
GO:0043523	ATM,BCL2,CASP7,CTNNB1,RIPK1,AIFM1
hsa05132	BCL2,CASP7,CTNNB1,MYD88,PIK3C2A,RAB5C,TCF7L2,RIPK1,ABI1,TUBB1,ADRB1,KCNH2,KPNB1,SOCS7,AFAP1L2,MPP7
hsa05132	BCL2,CASP7,CTNNB1,MYD88,PIK3C2A,RAB5C,TCF7L2,RIPK1,ABI1,TUBB1
GO:0019904	ADRB1,BCL2,CTNNB1,KCNH2,KPNB1,MYD88,TCF7L2,RIPK1,ABI1,SOCS7,AFAP1L2,MPP7
GO:0048762	BCL2,CTNNB1,EDNRB,FOXF2,FOXC1,STAT1,SP6,AMER1,ACAT1,AQP1,GFRA1,CCR7,TCF7L2,ATM,TDRD1,BMP15,MASTL,NKAP,KRT17,NSDHL,POU4F3,ZEB1,MYO3B,GRM6,KRT12,VPS4B,CDX4
GO:0048762	BCL2,CTNNB1,EDNRB,FOXF2,FOXC1,STAT1,SP6,AMER1
GO:0072009	ACAT1,AQP1,BCL2,CTNNB1,EDNRB,FOXC1
GO:0001822	ACAT1,AQP1,BCL2,CTNNB1,EDNRB,FOXC1,GFRA1,STAT1,AMER1
GO:0001656	ACAT1,AQP1,BCL2,CTNNB1,STAT1
GO:0060485	BCL2,CTNNB1,EDNRB,FOXF2,FOXC1,STAT1,SP6,AMER1
GO:0072001	ACAT1,AQP1,BCL2,CTNNB1,EDNRB,FOXC1,GFRA1,STAT1,AMER1
GO:0061326	ACAT1,AQP1,BCL2,CTNNB1,STAT1
GO:0072006	ACAT1,AQP1,BCL2,CTNNB1,EDNRB,FOXC1
GO:0072073	ACAT1,AQP1,BCL2,CTNNB1,EDNRB,FOXC1
GO:0010560	CCR7,CTNNB1,TCF7L2
GO:0048599	ATM,BCL2,CTNNB1,TDRD1
GO:0010559	BCL2,CCR7,CTNNB1,TCF7L2
GO:0009994	ATM,BCL2,CTNNB1,TDRD1
GO:0032835	AQP1,BCL2,EDNRB,FOXC1
GO:1903020	CCR7,CTNNB1,TCF7L2
GO:1903018	BCL2,CCR7,CTNNB1,TCF7L2
GO:0007292	ATM,BCL2,CTNNB1,BMP15,TDRD1,MASTL
GO:0033077	BCL2,CCR7,CTNNB1,NKAP
GO:0031069	BCL2,CTNNB1,KRT17
GO:0048730	BCL2,CTNNB1,KRT17
GO:0001942	BCL2,CTNNB1,KRT17,NSDHL
GO:0022404	BCL2,CTNNB1,KRT17,NSDHL
GO:0022405	BCL2,CTNNB1,KRT17,NSDHL
GO:0072080	ACAT1,AQP1,BCL2,CTNNB1

GO:0090596	AQP1,BCL2,CTNNB1,FOXF2,POU4F3,ZEB1,MYO3B
GO:0061005	EDNRB,STAT1,AMER1
GO:0042303	BCL2,CTNNB1,KRT17,NSDHL
GO:0042633	BCL2,CTNNB1,KRT17,NSDHL
GO:0048538	ATM,BCL2,CTNNB1
hsa05215	BCL2,CTNNB1,TCF7L2,ZEB1
GO:0048592	AQP1,BCL2,CTNNB1,FOXF2,ZEB1
GO:0001654	AQP1,BCL2,CTNNB1,FOXF2,FOXC1,GRM6,KRT12,ZEB1
GO:0048477	ATM,BCL2,CTNNB1,TDRD1
GO:0060070	CTNNB1,EDNRB,TCF7L2,VPS4B
GO:0150063	AQP1,BCL2,CTNNB1,FOXF2,FOXC1,GRM6,KRT12,ZEB1
GO:0048880	AQP1,BCL2,CTNNB1,FOXF2,FOXC1,GRM6,KRT12,ZEB1
GO:0009948	CDX4,CTNNB1,TDRD1
GO:0007423	AQP1,BCL2,CTNNB1,FOXF2,FOXC1,GRM6,KRT12,POU4F3,ZEB1,MYO3B
GO:0098773	BCL2,CTNNB1,KRT17,NSDHL
GO:0016301	ATM,MAP3K8,FLT4,PHKA2,PIK3C2A,PIP4K2A,TK2,DGKD,RIPK1,OXSR1,MAP3K2,MASTL,ATP23,MYO3B,PGM2L1,MYLK4,KAT2A,P3H4,ZDHHC9,POGLUT2
GO:0016301	ATM,MAP3K8,FLT4,PHKA2,PIK3C2A,PIP4K2A,TK2,DGKD,RIPK1,OXSR1,MAP3K2,MASTL,ATP23,MYO3B,PGM2L1,MYLK4
GO:0016773	ATM,MAP3K8,FLT4,PHKA2,PIK3C2A,PIP4K2A,DGKD,RIPK1,OXSR1,MAP3K2,MASTL,ATP23,MYO3B,PGM2L1,MYLK4
GO:0004674	ATM,MAP3K8,PHKA2,RIPK1,OXSR1,MAP3K2,MASTL,ATP23,MYO3B,MYLK4
GO:0016310	ATM,MAP3K8,FLT4,PIK3C2A,PIP4K2A,TK2,DGKD,RIPK1,OXSR1,MAP3K2,MASTL,MYO3B,MYLK4
GO:0004672	ATM,MAP3K8,FLT4,PHKA2,RIPK1,OXSR1,MAP3K2,MASTL,ATP23,MYO3B,MYLK4
GO:0018193	ATM,FLT4,KAT2A,RIPK1,OXSR1,P3H4,ZDHHC9,POGLUT2,MASTL,MYO3B
GO:0106310	ATM,MAP3K8,RIPK1,OXSR1,MAP3K2,MASTL,MYO3B,MYLK4
GO:0018209	ATM,RIPK1,POGLUT2,MASTL,MYO3B
hsa04070	OCRL,PIK3C2A,PIP4K2A,DGKD,MTMR7,PLCD4,ATM,SOCS7,ACAT1,ACLY,KDSR,PDSS1,NSDHL,OSBPL7,GARS1,TK2,SLC22A13
hsa04070	OCRL,PIK3C2A,PIP4K2A,DGKD,MTMR7,PLCD4
GO:0046854	ATM,OCRL,PIK3C2A,PIP4K2A,SOCS7
hsa00562	OCRL,PIK3C2A,PIP4K2A,MTMR7,PLCD4
GO:0001727	ATM,PIK3C2A,PIP4K2A,DGKD
GO:0006661	ATM,OCRL,PIK3C2A,PIP4K2A,MTMR7,SOCS7
GO:0008610	ACAT1,ACLY,ATM,KDSR,OCRL,PIK3C2A,PIP4K2A,DGKD,MTMR7,PDSS1,SOCS7,NSDHL,OSBPL7
GO:0036092	ATM,OCRL,PIK3C2A

GO:0090407	ACAT1,ACLY,ATM,GARS1,OCRL,PIK3C2A,PIP4K2A,TK2,DGKD,MTMR7,SLC22A13,SOCS7
GO:0046474	ATM,OCRL,PIK3C2A,PIP4K2A,DGKD,MTMR7,SOCS7
GO:0052742	ATM,PIK3C2A,PIP4K2A
GO:0046488	ATM,OCRL,PIK3C2A,PIP4K2A,MTMR7,SOCS7
GO:0045017	ATM,OCRL,PIK3C2A,PIP4K2A,DGKD,MTMR7,SOCS7
GO:0008654	ATM,OCRL,PIK3C2A,PIP4K2A,DGKD,MTMR7,SOCS7
GO:0006650	ATM,OCRL,PIK3C2A,PIP4K2A,DGKD,MTMR7,SOCS7
GO:0019903	BCL2,CDH5,CTNNB1,FLT4,KAT2A,STAT1,MASTL,STAT5A,RIPK1,AIFM1,VPS4B,PLCD4,KCNH2,CUL5,RPL23
GO:0019903	BCL2,CDH5,CTNNB1,FLT4,KAT2A,STAT1,MASTL
GO:0019902	BCL2,CDH5,CTNNB1,FLT4,KAT2A,STAT1,MASTL
hsa04217	BCL2,STAT1,STAT5A,RIPK1,AIFM1,VPS4B
GO:0051721	BCL2,STAT1,MASTL
hsa04933	BCL2,STAT1,STAT5A,PLCD4
GO:0044389	BCL2,CTNNB1,KCNH2,STAT1,CUL5,RIPK1,RPL23
GO:0043296	ADCYAP1R1,CDH5,CTNNB1,PLXDC1,SHROOM4,MARVELD3,MPP7,CDH10,GFRA1,MYD88,PIK3C2A,SOCS7,XIRP2,JCAD
GO:0043296	ADCYAP1R1,CDH5,CTNNB1,PLXDC1,SHROOM4,MARVELD3,MPP7
GO:0019898	CDH5,CDH10,CTNNB1,GFRA1,MYD88,PIK3C2A,SOCS7
GO:0005923	ADCYAP1R1,CDH5,CTNNB1,PLXDC1,MARVELD3,MPP7
GO:0070160	ADCYAP1R1,CDH5,CTNNB1,PLXDC1,MARVELD3,MPP7
GO:0016342	CDH5,CDH10,CTNNB1
GO:0045216	CDH5,CDH10,CTNNB1,MARVELD3,XIRP2,MPP7
GO:0007043	CDH5,CDH10,CTNNB1,MARVELD3,MPP7
GO:0005912	CDH5,CDH10,CTNNB1,SHROOM4,JCAD,MPP7
GO:0070830	CDH5,MARVELD3,MPP7
GO:0034332	CDH5,CDH10,CTNNB1
GO:0019897	CDH5,CDH10,CTNNB1,MYD88
GO:0120192	CDH5,MARVELD3,MPP7
GO:0043297	CDH5,MARVELD3,MPP7
GO:0120193	CDH5,MARVELD3,MPP7
GO:0060322	AQP1,ATM,BCL2,CNP,CTNNB1,DDX10,FOXC1,KAT2A,ZEB1,TOP2B,SLC38A2,ODAD2,SHROOM4,GPR158,UPF3B,ODAD4,CDX4,PCGF2,ABI1,TDRD1,NPAT,OCRL,NSDHL,KIAA1217,CMTM3,FOXF2,POU4F3,MYO3B,ARID2,CASP7,NEBL,XIRP2
GO:0060322	AQP1,ATM,BCL2,CNP,CTNNB1,DDX10,FOXC1,KAT2A,ZEB1,TOP2B,SLC38A2,ODAD2,SHROOM4,GPR158,UPF3B,ODAD4
GO:0009952	ATM,CDX4,CTNNB1,FOXC1,KAT2A,PCGF2,ABI1,TDRD1

GO:0007420	AQP1,ATM,BCL2,CNP,CTNNB1,FOXC1,KAT2A,ZEB1,TOP2B,SLC38A2,ODAD2,SHROOM4,GPR158,UPF3B,ODAD4
GO:0007389	ATM,CDX4,CTNNB1,FOXC1,KAT2A,ZEB1,PCGF2,ABI1,ODAD2,TDRD1,ODAD4
GO:0035282	ATM,FOXC1,KAT2A,ABI1,TDRD1
GO:0003002	ATM,CDX4,CTNNB1,FOXC1,KAT2A,PCGF2,ABI1,ODAD2,TDRD1,ODAD4
GO:0043009	ATM,CDX4,CTNNB1,FOXC1,KAT2A,NPAT,OCRL,ZEB1,PCGF2,ABI1,NSDHL,KIAA1217,CMTM3
GO:0001756	ATM,FOXC1,KAT2A,ABI1
GO:0009792	ATM,CDX4,CTNNB1,FOXC1,KAT2A,NPAT,OCRL,ZEB1,PCGF2,ABI1,NSDHL,KIAA1217,CMTM3
GO:0048568	CDX4,CTNNB1,FOXF2,FOXC1,POU4F3,ZEB1,PCGF2,NSDHL,MYO3B,ARID2
GO:0001701	CDX4,CTNNB1,FOXC1,KAT2A,NPAT,OCRL,PCGF2,NSDHL,CMTM3
GO:0061053	ATM,FOXC1,KAT2A,ABI1
GO:0007507	ATM,CASP7,CTNNB1,FOXC1,KAT2A,NEBL,ODAD2,ODAD4,XIRP2,ARID2
GO:0006970	AQP1,TSC22D3,SERPINB6,OXSR1,MARVELD3,EDNRB,MYD88,MAP3K2,SLC38A2,CTNNB1,STAT1,ATM
GO:0006970	AQP1,TSC22D3,SERPINB6,OXSR1,MARVELD3
GO:0070293	AQP1,EDNRB,OXSR1
GO:0071260	AQP1,MYD88,MAP3K2,SLC38A2
GO:0071470	AQP1,SERPINB6,OXSR1
GO:0009612	AQP1,CTNNB1,MYD88,STAT1,MAP3K2,SLC38A2
GO:0071214	AQP1,ATM,MYD88,SERPINB6,OXSR1,MAP3K2,SLC38A2
GO:0104004	AQP1,ATM,MYD88,SERPINB6,OXSR1,MAP3K2,SLC38A2
GO:0005635	AQP1,BCL2,CDH5,CETN2,EDNRB,KPNB1,VPS4B,SCRN1,RBMX2,GHDC,OSBPL7,CMTM3
GO:0005635	AQP1,BCL2,CDH5,CETN2,EDNRB,KPNB1,VPS4B,SCRN1,RBMX2,GHDC,OSBPL7,CMTM3
GO:0031965	AQP1,BCL2,CDH5,EDNRB,KPNB1,SCRN1,RBMX2,OSBPL7,CMTM3
GO:0061458	ATM,BCL2,CTNNB1,FOXF2,FOXC1,GFRA1,SERPINB5,PLEKHA5,KLHL10,ADCYAP1R1,PIK3C2A,PIP4K2A,RRAGB,MID2,WAC,EXOC1,OSBPL7,SMARCE1,VPS4B,CTDSPL,AFAP1L2,MASTL,ARID2,SASH3,CDH5
GO:0061458	ATM,BCL2,CTNNB1,FOXF2,FOXC1,GFRA1,SERPINB5,PLEKHA5,KLHL10
GO:0007548	ADCYAP1R1,ATM,BCL2,CTNNB1,FOXF2,FOXC1,GFRA1,KLHL10
GO:0010506	ATM,BCL2,PIK3C2A,PIP4K2A,RRAGB,MID2,WAC,EXOC1,OSBPL7
GO:0048608	ATM,BCL2,CTNNB1,FOXF2,FOXC1,GFRA1,SERPINB5,KLHL10
GO:0007346	ATM,BCL2,CTNNB1,FOXC1,SMARCE1,VPS4B,CTDSPL,WAC,AFAP1L2,MASTL,ARID2
GO:0048873	BCL2,SASH3,KLHL10
GO:2000045	ATM,BCL2,SMARCE1,CTDSPL,WAC,ARID2
GO:2000134	ATM,BCL2,CTDSPL,WAC
GO:1902806	ATM,BCL2,SMARCE1,CTDSPL,WAC,ARID2

GO:1902807	ATM,BCL2,CTDSPL,WAC
GO:0001894	BCL2,CDH5,CTNNB1,FOXC1,SASH3,KLHL10
GO:0060249	BCL2,CDH5,CTNNB1,FOXC1,SASH3,KLHL10
GO:0046545	ADCYAP1R1,ATM,BCL2,FOXC1
GO:0045137	ADCYAP1R1,ATM,BCL2,FOXC1,GFRA1,KLHL10
GO:0001541	ATM,BCL2,FOXC1
GO:0045930	ATM,BCL2,CTNNB1,FOXC1,CTDSPL,WAC
GO:0046660	ADCYAP1R1,ATM,BCL2,FOXC1
GO:0005938	CTNNB1,GAD1,LASP1,SEPTIN6,EXOC1,SHROOM4,SCIN,CAPZA3,MPP7
GO:0005938	CTNNB1,GAD1,LASP1,SEPTIN6,EXOC1,SHROOM4,SCIN,CAPZA3,MPP7
GO:0032986	SMARCE1,RPL23,ARID2
GO:0032986	SMARCE1,RPL23,ARID2
GO:0046328	CCR7,FLT4,MYD88,RIPK1,DUSP22,MARVELD3,KAT2A,IRF4,STAT1,TBX21,SASH3,DHX58,AFAP1L2,EDNRB,MID2,NKIRAS2,CTNNB1,IGFBP4,JCAD,RELL1,MAP3K8,ABI1,MPP7,SPRY3
GO:0046328	CCR7,FLT4,MYD88,RIPK1,DUSP22,MARVELD3
GO:0046330	CCR7,FLT4,MYD88,RIPK1,DUSP22
GO:0001819	CCR7,FLT4,KAT2A,IRF4,MYD88,STAT1,RIPK1,TBX21,SASH3,DHX58,AFAP1L2
GO:0043122	CCR7,EDNRB,MYD88,STAT1,RIPK1,MID2,NKIRAS2
GO:0032813	MYD88,STAT1,RIPK1
GO:0043410	CCR7,CTNNB1,FLT4,IGFBP4,MYD88,RIPK1,DUSP22,JCAD,RELL1
hsa04620	MAP3K8,MYD88,STAT1,RIPK1
GO:0035591	MYD88,RIPK1,ABI1,MPP7
GO:0043408	CCR7,CTNNB1,FLT4,IGFBP4,MYD88,RIPK1,SPRY3,DUSP22,JCAD,MARVELD3,RELL1
GO:0042803	BCL2,FLT4,GRM6,KCNH2,NQO2,PIP4K2A,STAT1,DGKD,RIPK1,SLC25A14,VPS4B,MID2,MTPAP,ACOD1,MYD88,EXOC1,DHX58,TBX21
GO:0042803	BCL2,FLT4,GRM6,KCNH2,NQO2,PIP4K2A,STAT1,DGKD,RIPK1,SLC25A14,VPS4B,MID2,MTPAP,ACOD1
GO:0051607	BCL2,MYD88,STAT1,MID2,EXOC1,DHX58,ACOD1
GO:0009615	BCL2,MYD88,STAT1,MID2,TBX21,EXOC1,DHX58,ACOD1
GO:0050678	CTNNB1,EDNRB,FLT4,SERPINB5,STAT1,STAT5A,TCF7L2,ZEB1,JCAD,MARVELD3,AQP1,CASP7,CRHR2,PIK3C2A,TOP2B,AIFM1,RPL23,RRAGB,CDH5,FOXC1
GO:0050678	CTNNB1,EDNRB,FLT4,SERPINB5,STAT1,STAT5A,TCF7L2,ZEB1,JCAD,MARVELD3
GO:0010464	CTNNB1,STAT1,ZEB1
GO:1901699	AQP1,CASP7,CRHR2,CTNNB1,PIK3C2A,STAT1,STAT5A,ZEB1,TOP2B,AIFM1,RPL23,RRAGB
GO:0071417	AQP1,CASP7,CRHR2,CTNNB1,PIK3C2A,STAT1,STAT5A,ZEB1,TOP2B,RPL23,RRAGB
GO:0045601	CDH5,CTNNB1,ZEB1

GO:0030857	CTNNB1,STAT1,ZEB1
GO:0030856	CDH5,CTNNB1,FOXC1,STAT1,ZEB1
GO:0051015	LASP1,ABLIM1,NEBL,SHROOM4,SCIN,CAPZA3,XIRP2,DUSP22,ARHGAP21,MYO3B,TNS4
GO:0051015	LASP1,ABLIM1,NEBL,SHROOM4,SCIN,CAPZA3,XIRP2
GO:0001725	ABLIM1,NEBL,SHROOM4,XIRP2
GO:0097517	ABLIM1,NEBL,SHROOM4,XIRP2
GO:0015629	LASP1,ABLIM1,NEBL,DUSP22,SHROOM4,ARHGAP21,SCIN,CAPZA3,XIRP2,MYO3B
GO:0003779	LASP1,ABLIM1,NEBL,SHROOM4,TNS4,SCIN,CAPZA3,XIRP2,MYO3B
GO:0042641	ABLIM1,NEBL,SHROOM4,XIRP2
GO:0032432	ABLIM1,NEBL,SHROOM4,XIRP2
GO:0000165	MAP3K8,CTNNB1,IGFBP4,MYD88,RIPK1,MAP3K2,ZDHHC9
GO:0000165	MAP3K8,CTNNB1,IGFBP4,MYD88,RIPK1,MAP3K2,ZDHHC9
GO:0004709	MAP3K8,RIPK1,MAP3K2
